# Identification of adult spinal Shox2 neuronal subpopulations based on unbiased computational clustering of electrophysiological properties

**DOI:** 10.3389/fncir.2022.957084

**Published:** 2022-08-04

**Authors:** D. Leonardo Garcia-Ramirez, Shayna Singh, Jenna R. McGrath, Ngoc T. Ha, Kimberly J. Dougherty

**Affiliations:** Department of Neurobiology and Anatomy, Marion Murray Spinal Cord Research Center, Drexel University College of Medicine, Philadelphia, PA, United States

**Keywords:** spinal cord, interneuron, locomotion, cluster analysis, electrophysiology

## Abstract

Spinal cord neurons integrate sensory and descending information to produce motor output. The expression of transcription factors has been used to dissect out the neuronal components of circuits underlying behaviors. However, most of the canonical populations of interneurons are heterogeneous and require additional criteria to determine functional subpopulations. Neurons expressing the transcription factor Shox2 can be subclassified based on the co-expression of the transcription factor Chx10 and each subpopulation is proposed to have a distinct connectivity and different role in locomotion. Adult Shox2 neurons have recently been shown to be diverse based on their firing properties. Here, in order to subclassify adult mouse Shox2 neurons, we performed multiple analyses of data collected from whole-cell patch clamp recordings of visually-identified Shox2 neurons from lumbar spinal slices. A smaller set of Chx10 neurons was included in the analyses for validation. We performed k-means and hierarchical unbiased clustering approaches, considering electrophysiological variables. Unlike the categorizations by firing type, the clusters displayed electrophysiological properties that could differentiate between clusters of Shox2 neurons. The presence of clusters consisting exclusively of Shox2 neurons in both clustering techniques suggests that it is possible to distinguish Shox2^+^Chx10^−^ neurons from Shox2^+^Chx10^+^ neurons by electrophysiological properties alone. Computational clusters were further validated by immunohistochemistry with accuracy in a small subset of neurons. Thus, unbiased cluster analysis using electrophysiological properties is a tool that can enhance current interneuronal subclassifications and can complement groupings based on transcription factor and molecular expression.

## Introduction

The spinal neuronal circuitry participates in the control of a wide variety of movements, ranging from reflexes to highly sophisticated motor skills (Hultborn and Nielsen, 2007; Goulding, 2009; Todd, 2010; Levine et al., 2012; Kiehn, 2016; Grillner and El Manira, 2020). The identification and connectivity of spinal interneuron populations that underlie specific behaviors is essential for a complete understanding of motor function. Spinal neuronal populations are highly diverse and functional populations are anatomically intermingled (Goulding, 2009; Kiehn, 2016; Abraira et al., 2017; Stachowski and Dougherty, 2021). The identification of spinal interneuronal populations based on the expression of transcription factors has defined neurons involved in or essential to rhythm generation, left-right alternation, flexor-extensor alternation, and patterning (Wilson et al., 2005; Gosgnach et al., 2006; Crone et al., 2008; Zhang et al., 2008, 2014; Dyck et al., 2012; Dougherty et al., 2013; Talpalar et al., 2013; Caldeira et al., 2017). However, each of the transcription factor-defined canonical classes of ventral interneurons identified to date is heterogeneous and serves multiple functions (Pierani et al., 2001; Brownstone and Wilson, 2008; Borowska et al., 2013; Bikoff et al., 2016; Shevtsova and Rybak, 2016; Hayashi et al., 2018; Deska-Gauthier et al., 2020; Falgairolle and O'Donovan, 2021).

As with other genetically identified populations, lumbar spinal cord neurons that express the transcription factor Shox2 are heterogeneous in transcription factor expression, connectivity, and function (Dougherty et al., 2013; Ha and Dougherty, 2018). Shox2 neurons overlap with the V2a (Chx10-expressing) population of neurons (Dougherty et al., 2013). Similarly, V2a neurons are also heterogeneous based on molecular expression (Song et al., 2009; Hayashi et al., 2018), firing types (Dougherty and Kiehn, 2010; Zhong et al., 2010; Husch et al., 2015), activity pattern during locomotor deletions (Zhong et al., 2012), and function (Crone et al., 2008, 2009). There is also interrelation between subpopulations. The Shox2 subpopulation lacking Chx10 expression has been linked to the generation of the locomotor rhythm (Dougherty et al., 2013; Dougherty and Ha, 2019), the Chx10^+^ neuronal population has been implicated in the left-right alternation (Crone et al., 2008, 2009), and the Shox2^+^Chx10^+^ neurons are thought to be pre-motoneurons participating in the stabilization of the locomotor burst (Dougherty et al., 2013).

Particularly for transcription factors that are developmentally downregulated or with deletions that are lethal, the relation of population to function comes largely from studies in neonates (Gosgnach et al., 2006; Crone et al., 2008; Zhang et al., 2008; Dougherty et al., 2013; Talpalar et al., 2013), as do experiments relating cellular firing or phasing during locomotor-like activity (Dougherty and Kiehn, 2010; Zhong et al., 2011; Dyck et al., 2012). Other classification schemes used to subdivide potential functional populations in both neonate and adult include location (Harrison et al., 1986; Puskar and Antal, 1997; Petko et al., 2004; Jankowska, 2008), neurotransmitter phenotype (Huang et al., 2000; Goulding et al., 2014; Bikoff et al., 2016; Hughes and Todd, 2020; Stachowski and Dougherty, 2021), morphology (Gobel, 1975; Maxwell et al., 2007; Yasaka et al., 2010), electrophysiological properties (Konnerth et al., 1990; Lopez-Garcia and King, 1994; Grudt and Perl, 2002; Dai et al., 2009; Dyck et al., 2012) and molecular profiles (Goulding, 2009; Kiehn, 2016; Ziskind-Conhaim and Hochman, 2017; Haring et al., 2018; Gatto et al., 2019). Recent RNA sequence profiling of adult spinal neurons has suggested clusters of spinal interneurons where ventral clusters outnumber the canonical V0–V3 developmentally-derived populations and some clusters contain significant numbers of neurons in more than one canonical population (Sathyamurthy et al., 2018). Similarly, mature neurons may be more diverse than those of neonate electrophysiologically and morphologically (Alvarez et al., 2005; Al-Mosawie et al., 2007). Electrophysiological recordings have demonstrated that adult Shox2 neurons display heterogeneous electrophysiological properties (Garcia-Ramirez et al., 2021), suggesting the possibility of more than two subpopulations. Electrophysiological properties establish the ways in which neurons respond to the synaptic inputs and neuromodulation, determine integration capacity, and shape output of individual neurons; therefore, along with connectivity, they govern spinal circuit function (Konnerth et al., 1990; Russo and Hounsgaard, 1999; Lee and Heckman, 2000; Butt et al., 2002; Miles et al., 2005; Smith and Perrier, 2006; Brownstone and Wilson, 2008; Dai et al., 2009; Grillner and El Manira, 2020). Thus, classification of neurons based on their passive and active properties can provide essential characteristics that should correlate with their participation in behavior.

Here, rather than constraining subpopulations of Shox2 neurons based on the expression of a second transcription factor or other molecular marker, we use electrophysiological properties to define subpopulations and then determine how electrophysiolocially-defined subpopulations relate to transcription factor-defined subdivisions. Using neuronal firing type and two clustering approaches, *k*-means clustering and hierarchical clustering, we identify the electrophysiological parameters that are characteristic to each subpopulation of Shox2 neurons. We found 4 populations of adult Shox2 neurons based on firing type, all of which were in common with Chx10 neurons. Analysis performed on 4 *k*-means clusters and 6 hierarchical clusters of Shox2 neurons identified clusters which overlap with Chx10 neurons to varying degrees. In both computational analyses, we found clusters that included Shox2 neurons but not Chx10 neurons, suggesting the possibility to identify Shox2^+^Chx10^−^ neurons with a single lineage traced mouse line. Unbiased computational analysis is a useful tool to identify populations of neurons in adult mice where electrophysiological diversity is high and extends beyond current molecular markers and transgenic tools. Further, results could have important implications for the study of neurons in intact preparations where the ability to visually identify neurons is limited.

## Materials and methods

Experiments were performed using 51 Shox2::Cre; Rosa26-flox-Stop-flox-tdTomato (Ai9 from Jax mice, #007909) transgenic mice (Madisen et al., 2010; Dougherty et al., 2013) and 6 Chx10-eGFP (from MMRRC, Cat#:011391-UCD) transgenic mice. Electrophysiological properties from 60 of the Shox2 neurons (from 28 of the mice) included in the new analyses were reported in a previous study (Garcia-Ramirez et al., 2021). All experimental procedures followed National Institutes of Health guidelines and were approved by the Institutional Animal Care and Use Committee at Drexel University.

### Spinal cord preparations

For terminal electrophysiology experiments, adult mice (8–25 weeks postnatal) were anesthetized with ketamine (150 mg/kg) and xylazine (15 mg/kg), decapitated, and eviscerated. Spinal cords were then removed in ice-cold dissecting solution containing the following (in mM): 222 glycerol, 3 KCl, 11 glucose, 25 NaHCO_3_, 1.3 MgSO_4_, 1.1 KH_2_PO_4_, and 2.5 CaCl_2_ and gassed with 95% O_2_ and 5% CO_2_ with pH adjusted to 7.4. The lumbar spinal cord (segments L2–5) was sectioned transversely using a vibrating microtome (Leica Microsystems). Slices were next transferred to an artificial cerebrospinal fluid, containing the following (in mM): 111 NaCl, 3 KCl, 11 glucose, 25 NaHCO_3_, 1.3 MgSO_4_, 1.1 KH_2_PO_4_, and 2.5 CaCl_2_ at 37°C for 30 min and then passively equilibrated to room temperature for another 30 min before recording. Dissecting and recording solutions were continuously aerated with 95%/5% O_2_/CO_2_.

### Whole-cell patch clamp recordings

All recordings were performed at room temperature. Fluorescently labeled (tdTomato) Shox2 and (eGFP) Chx10 neurons were visualized with a 63x objective lens on a BX51WI scope (Olympus) using LED illumination (Andor Mosaic System or Lumen Dynamics X-Cite 120 LED). Patch electrodes were pulled to tip resistances of 5–8 MΩ using a multistage puller (Sutter Instruments) and were filled with intracellular solution, which contained the following (in mM): 128 K-gluconate, 10 HEPES, 0.0001 CaCl_2_, 1 glucose, 4 NaCl, 5 ATP, and 0.3 GTP, with pH adjusted to 7.4. In some experiments, biocytin (2 mg/ml, B4261, Sigma-Aldrich) was included in the patch electrode. Data were collected with a Multiclamp 700B amplifier (Molecular Devices) and Clampex software (pClamp9, Molecular Devices). Signals were digitized at 20 kHz and filtered at 10 kHz.

### Measurement and calculation of passive and active membrane properties

**Figure 1:**
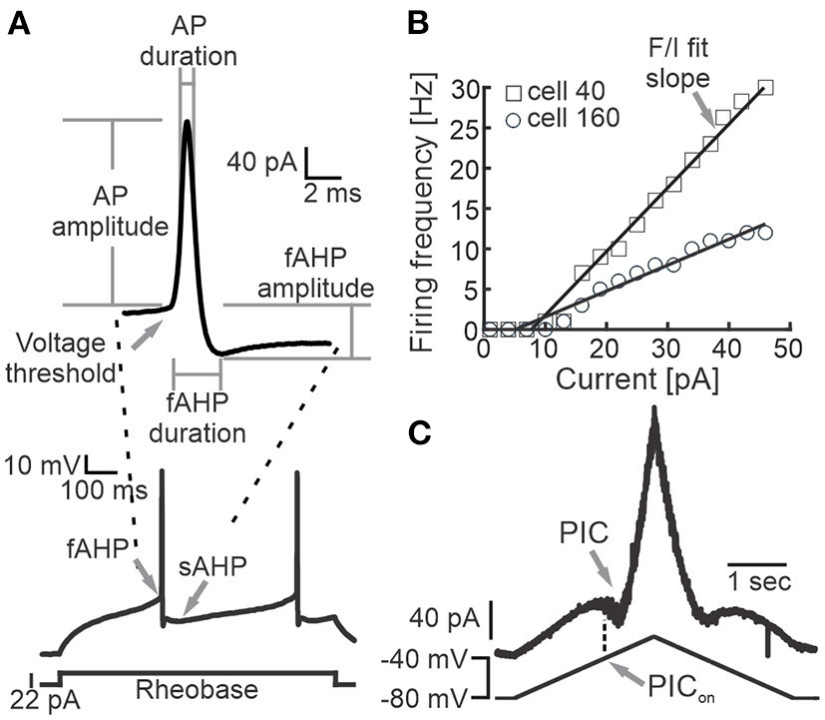
Measured electrophysiological properties. **(A)** Representative example of a recording of the membrane potential of a Shox2 neuron during the injection of a 1 s long current step at rheobase (bottom) and magnification of the first action potential generated (top). Arrows point out the voltage threshold and fast and slow AHP. The AP and fAHP amplitude and duration measurement points are shown. **(B)** Action potential firing frequency in response to the injection of depolarizing currents in two representative Shox2 neurons (squares, cell #40; circles, cell #160). Lines represent the F/I fit slope (arrow). **(C)** Current response (upper trace) to slow depolarizing ramp injection. Arrows point out the presence of PIC and the corresponding voltage on the ramp to activate the PIC (PIC_on_).

We performed whole-cell patch clamp recordings from Shox2 neurons and Chx10 neurons identified by fluorescence expression. Following exclusion of neurons with a resting membrane potential more depolarized than −40 mV and neurons with an action potential peak that did not reach 0 mV, 143 Shox2 neurons and 28 Chx10 neurons were considered for this study. Passive and active membrane properties were recorded or calculated as described briefly here. Current step protocols were run when neurons were at ~-70 mV and in most cases this required bias current. Resting membrane potential (E_m_) was recorded shortly after entering whole-cell mode. Input resistance (R_in_) was calculated from the current/voltage slope in response to hyperpolarizing voltage steps in which no voltage-gated current activation was evident. Membrane time constant (τ) was calculated as the time to reach 63% of the maximum depolarization in response to a subthreshold depolarizing current step. Membrane capacitance (C_m_) was calculated from the time constant and input resistance (C_m_ = R_in_/τ). Rheobase was the minimal current step required to generate an action potential, applied in intervals of 2–3pA. Additionally, we recorded action potential (AP) properties from the first AP observed in the depolarizing current at rheobase (Figure 1A). AP voltage threshold was the membrane potential at the first deflection of the AP. AP half width (AP duration) was determined at the voltage midway from the AP threshold to the peak. The fast afterhyperpolarization (fAHP) amplitude was the difference between the AP threshold and the maximum hyperpolarization. We normalized the fAHP amplitude by dividing by the AP amplitude. The durations of the fAHP and slow afterhyperpolarization (sAHP) were measured by the time elapsed from the AP threshold to the peak fast or slow hyperpolarization. For neurons firing with an initial double or initial burst, the slow and fast AHP values were measured from the AP threshold of the last AP of the double or burst. AP frequency was calculated as the number of action potentials in response to a 1 second depolarizing current injection at 1.5x rheobase. Frequency-current (F/I) curve slope was obtained from the number of APs generated by the administration of depolarizing current pulses from 1 to 49 pA at intervals of 3pA (Figure 1B). The AP frequency at resting membrane potential was calculated from the number of APs recorded in 1 min. We also recorded the voltage onset of the persistent inward currents (PIC_on_ voltage, Figure 1C), measured as the membrane potential at which a negative deflection began during the application of a slow (28 mV/s) voltage ramp.

### Statistical analyses

Data analysis was performed with Clampfit (Molecular Devices) and MATLAB (MathWorks). Statistical tests and *post-hoc* analyses used are stated for each experiment and performed with GraphPad Prism (GraphPad Software) and MATLAB. All results are presented as mean ± SD. Statistical significance was set at *p* < 0.05 unless otherwise stated. The distribution of the data was determined by Shapiro–Wilk normality test. The statistical comparisons between Shox2 and Chx10 neurons were performed by Mann-Whitney test or unpaired *t-*test. Comparisons between clusters were performed by Kruskal-Wallis with Dunn's *post-hoc* test or repeated-measures one-way ANOVA with Bonferroni *post-hoc* test. Comparisons between percentages were performed by chi-square test.

### Correlations, principal component analysis, and cluster analysis

**Figure 2:**
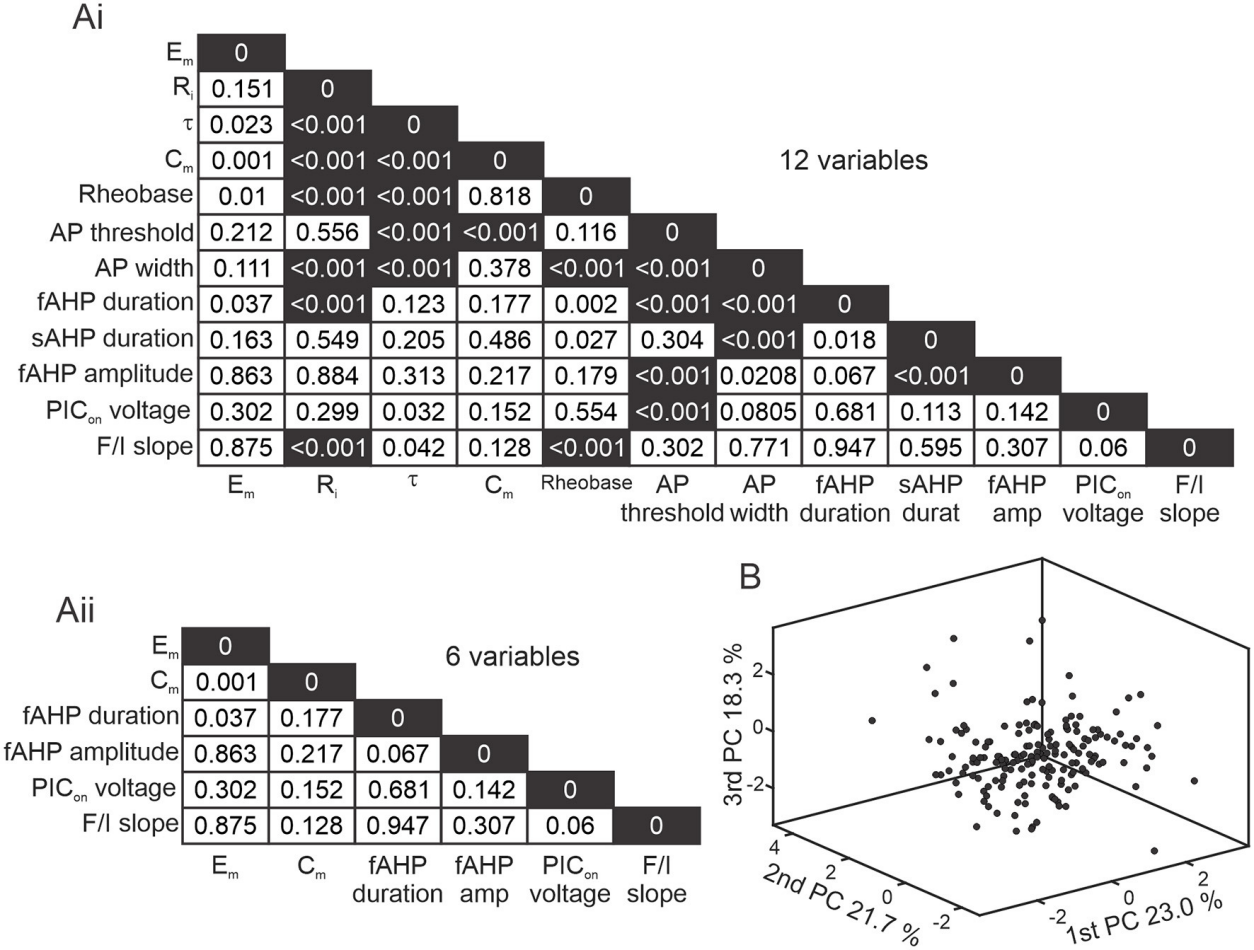
Reducing dimensionality of electrophysiological properties. **(A)** Correlation matrix of *p*-values from a Pearson's linear correlation of 12 **(Ai)** and 6 **(Aii)** electrophysiological properties from 143 Shox2 and 28 Chx10 neurons. Dark shading corresponds to highly correlated values (*p* < 0.001). **(B)** Three-dimensional representation of all 171 neurons included (black dots) after performing principal component analysis (PCA). X axis is the 1^st^ principal component (PC), Y axis is the 2^nd^ PC, and Z axis is the 3^rd^ PC, representing 23.0%, 21.7% and 18.3% of the variability, respectively.

To determine correlations in between the 12 properties obtained for each cell, we performed a Pearson's linear correlation test and considered values of *p* < 0.01 to be correlated. Principal component analysis (PCA) and multidimensional cluster analyses were performed on the 6 parameters obtained that were not highly correlated. PCA was used to reduce dimensionality of the number of variables recorded (Figure 2). To visualize clustering results, we show 3D graphs displaying neurons or means of clusters in a 3-dimensional space formed by the 3 first PCAs that explained more than 63% of the variability of the data. We classified Shox2 and Chx10 neurons by 3 different methods: (1) Firing types were determined by responses to depolarizing current steps. (2) For *k*-means clustering, the number of clusters was determined by the elbow method on a silhouette value graph after iterating the algorithm 1,000 times considering k = 2 to k = 8 clusters. Details can be found in the results section. (3) Hierarchical clusters were determined by the cosine distance between pairs of observations and consideration of an average for the distance between clusters. This combination was used because it resulted in the highest cophenetic correlation coefficient. The number of clusters was determined considering the cutoff below the maximum inconsistency coefficient for each link. For the k-means and hierarchical cluster analyses, we applied MATLAB algorithms, initially standardizing the data to set a mean of 0 and standard deviation of 1 to be able to compare variables with different units.

### Immunostaining and visualization of biocytin-filled neurons

The slices containing biocytin-filled neurons were fixed overnight (4% paraformaldehyde in 0.1 M PBS; pH 7.4; 4°C), and subsequently placed in PBS at 4°C. To visualize biocytin, the slices were incubated for 2 h at room temperature in DyLight 633 conjugated streptavidin (21844, Thermo Fischer Scientific). Then, the slices were washed in PBS (3 x 10 min), permeated with 1% Bovine Serum Albumin (BSA), 5% Donkey Serum (NGS), 0.1% Fish Gelatin, and 0.2% Triton x-100 for 1 h and incubated in sheep anti-Chx10 antibody 1:100 (AB9016, Chemicon) at room temperature for 48 hrs. Slices were washed in PBS (3 x 10 min) and incubated for 2 h at room temperature with rabbit anti-sheep Dylight 488 1:400 (SA5-10054, Thermo Fischer Scientific) followed by a final wash in PBS (3 x 10 min). Slices were then placed on slides within tissue spacers and coverslipped with a Vectashield mounting medium (Vector labs). Sections were scanned using a laser scanning confocal microscope (Leica True Confocal System SP8) in stacks of 10 optical sections across approximately 50 μm at 20x magnification. Images were condensed into maximum projections using the Leica collection software and brightness and contrast were adjusted in ImageJ.

## Results

### Correlation of the electrophysiological properties of adult Shox2 and Chx10 neurons

Excitatory spinal neurons expressing the transcription factor Shox2 are heterogeneous in terms of firing properties (Garcia-Ramirez et al., 2021). Since the passive and active properties shape the recruitment of, integration of inputs to, and output of neurons (Wilson et al., 2007; Tazerart et al., 2008; Harris-Warrick, 2010; Dougherty and Ha, 2019; Grillner and El Manira, 2020), we hypothesized that these properties could be used to subclassify Shox2 neurons. Since the Chx10 neuronal population partly overlaps with Shox2 neurons and is also heterogeneous (Dougherty and Kiehn, 2010; Zhong et al., 2010, 2012; Dougherty et al., 2013; Husch et al., 2015) we included a smaller set of Chx10 neurons in our sample. We first considered passive and active membrane properties of Shox2 and Chx10 neurons (Figure 1) and performed a correlation analysis (Figure 2Ai) to eliminate redundancy in the parameters measured. We found high correlations (*p* < 0.001) between some passive and active cellular properties (Figure 2Ai black squares). We removed the variables that were highly correlated from the analysis, which left 6 variables that were not highly correlated: resting membrane potential (E_m_), capacitance (C_m_), fast afterhyperpolarization (fAHP) duration, fAHP amplitude, activation voltage of the persistent inward current (PIC_on_) and frequency-current (F/I) slope (Figure 2Aii). To visualize the neurons in a three-dimensional plane, we performed a principal component analysis (PCA) based on the six variables (Figure 2B). The first three principal components explained 63% of the variability of the 171 neurons. This analysis shows that dimensionality of the variables recorded can be reduced by removing highly correlated properties and considering only 6 neuronal properties.

### Electrophysiological comparison between Shox2 and Chx10 neuronal populations

**Figure 3:**
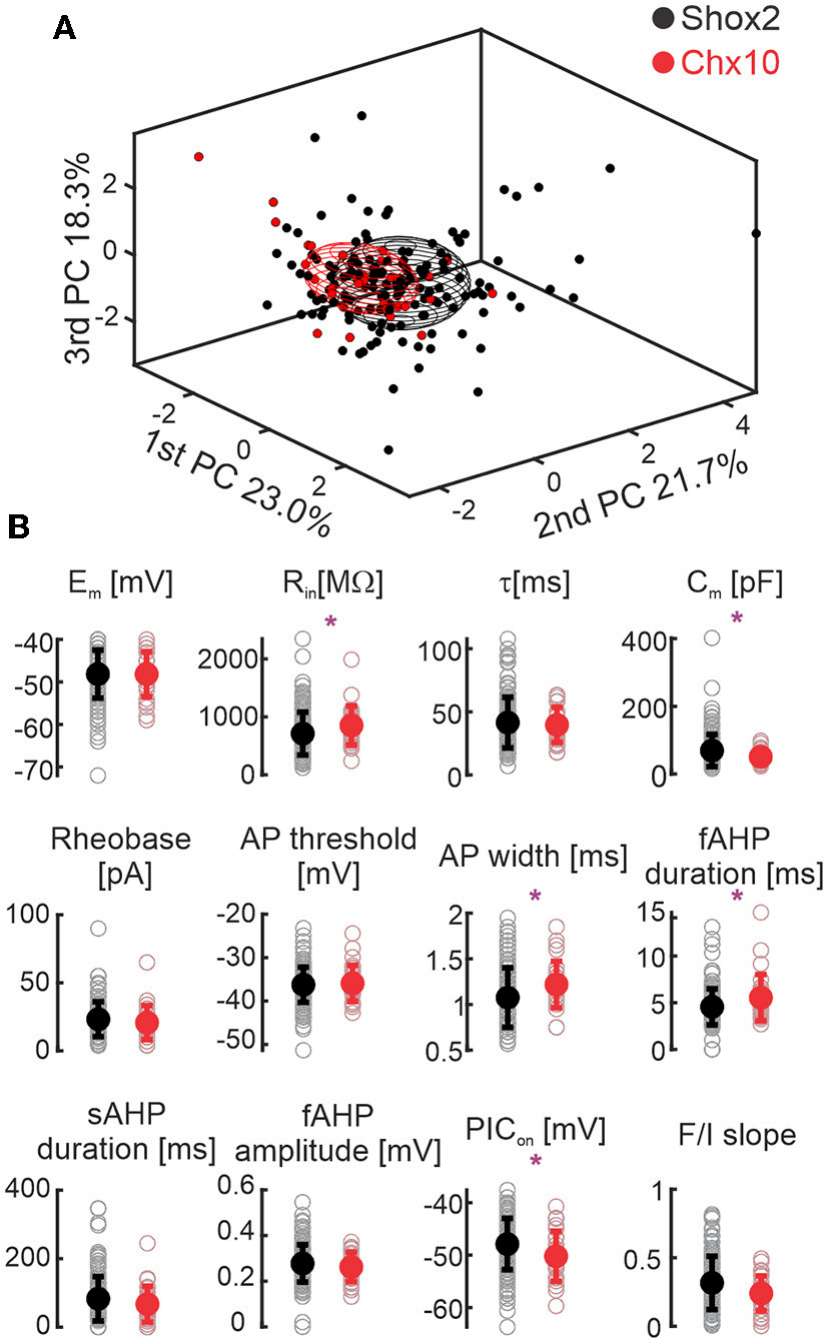
Comparison between Shox2 and Chx10 neurons. **(A)** Three-dimensional representation of 143 Shox2 neurons (black dots) and 28 Chx10 neurons (red dots) on the three principal components (same as Figure 2, but different angle). Ellipsoids are centered on the mean for the three first PCs and semi-axes are generated by the standard deviation of each of the three first PCs. Black and red ellipsoids represent groups of Shox2 and Chx10 neurons, respectively. **(B)** Comparison of electrophysiological properties measured from Shox2 neurons (black) and Chx10 neurons (red). Filled circles represent the mean and error bars the standard deviation, unfilled gray circles represent individual neurons. Resting membrane potential (E_m_), input resistance (R_in_), time constant (τ), calculated capacitance (C_m_), rheobase, action potential (AP) threshold, AP width, fast after hyperpolarization (fAHP) duration, slow afterhyperpolarization (sAHP) duration, fast after hyperpolarization (fAHP) amplitude, PIC activation voltage (PIC_on_), and slope of the AP frequency/current (F/I) curve. *p < 0.05, unpaired *t*-test or Mann-Whitney test.

Graphical display of the three first PCAs shows that Shox2 and Chx10 neurons partially overlap. We compared the initial 12 electrophysiological properties of adult Shox2 neurons (*n* = 143) with those of Chx10 neurons (*n* = 28) (Figure 3, Supplementary Table 1). Shox2 neurons had lower input resistance (R_in_), larger capacitance (C_m_), shorter AP half width, shorter fAHP duration, and more depolarized PIC activation voltages (PIC on) than Chx10 neurons. Resting membrane potential, time constant, rheobase, action potential threshold, the sAHP duration, fAHP amplitude, and F/I slope were similar between the groups. This comparison shows that Shox2 neurons and Chx10 neurons share some electrophysiological characteristics, however, differences are observed between subpopulations.

### Classification of the neurons based on firing type

**Figure 4:**
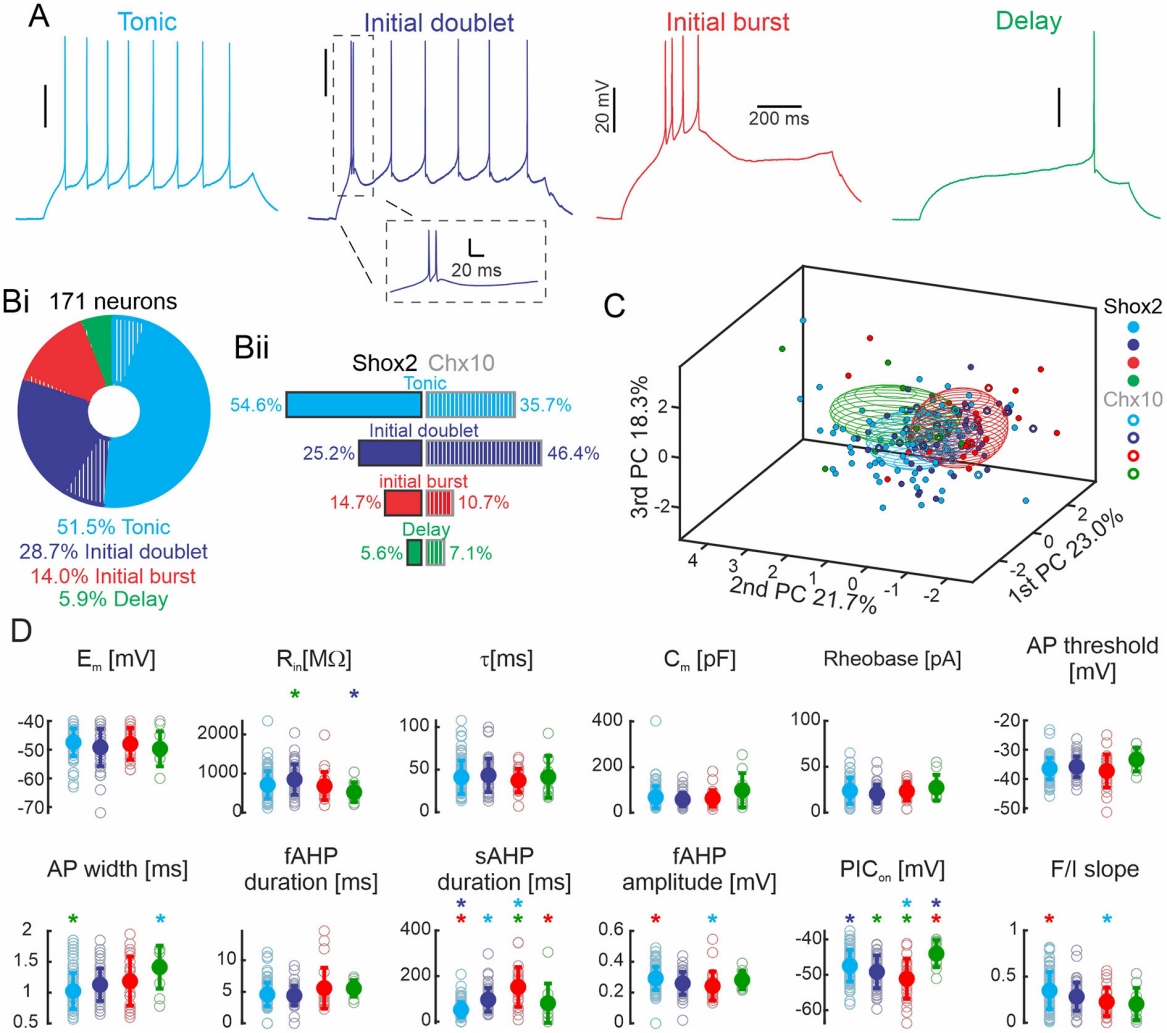
Shox2 and Chx10 neurons sorted based on type of firing. **(A)** Representative traces of the four action potential firing types in response to the injection of a 1-s depolarizing current step. **(Bi)** Pie chart and incidence of the four types of firing responses observed in all 171 neurons. 83.6% of the neurons were Shox2 neurons (solid) and 16.4% were Chx10 neurons (hatched). **(Bii)** Incidence of the four firing types observed in 143 Shox2 neurons (left) and in 28 Chx10 neurons (right). **(C)** Three-dimensional representation of all neurons (Shox2 filled dots, Chx10 unfilled dots) on the three principal component axes (same as Figure 2B, but different angle displayed). Ellipsoids are centered on the mean for the three first PCs and semi-axes are generated by the standard deviation of each of the three first PCs. Cyan, blue, red, and green ellipsoids represent tonic, initial doublet, initial burst, and delay firing subgroups, respectively. **(D)** Comparisons of electrophysiological properties of tonic, initial doublet, initial burst and delay firing neurons. Colors as in other panels. Filled circles represent the mean and error bars the standard deviation, unfilled gray circles represent individual neurons. Properties as in Figure 3. *p < 0.05, one-way ANOVA with Tukey *post-hoc* test or Kruskal-Wallis with Dunn's *post-hoc* test. * significantly different from the corresponding color coded group.

To identify differences in active and passive cellular properties based on firing type, we classified the 171 Shox2 and Chx10 neurons based on the response to suprathreshold depolarizing current steps. We found four types of responses (Figure 4A). Neurons firing throughout the current step were most prevalent. Over half of the neurons fired action potentials throughout and are referred to here as tonic firing neurons (Figure 4 cyan, *n* = 88, 51.5%). Initial doublet neurons (blue, *n* = 49, 28.7%) fire a doublet of action potentials at the start of the step (initial interspike interval <40 ms) and continue firing throughout the step but at a lower frequency (steady frequency of 3.9 ± 5.1 Hz). Neurons with burst of action potentials at the start of the current step are called initial burst firing neurons (red, *n* = 24, 14%). These neurons displayed three or more initial spikes at high frequency (>25 Hz) and were either silent or fired action potentials later in the step but these action potentials were not a regular frequency like the tonic neurons. Lastly, a small number of neurons, delay neurons (green, *n* = 10, 5.9%) fired action potentials after a delay from the beginning of the current step. We next looked at the prevalence of firing types in Shox2 and Chx10 neuronal populations. Of the 143 Shox2 neurons, tonic firing neurons were most common (*n* = 78, 54.6%), followed by initial doublet (*n* = 36, 25.2%) and initial burst (*n* = 21, 14.7%) firing, with delayed firing neurons (*n* = 8, 5.6%) being rare (Figure 4Bii, left side). Of the 28 Chx10 neurons, initial doublet firing neurons were most prevalent (*n* = 13, 46.4%), over one third were tonic firing (*n* = 10, 35.7%), and few neurons fired with an initial burst (*n* = 3, 10.7%) or were delayed firing (*n* = 2, 7.1%, Figure 4Bii, right side). The difference in the distribution of the 4 firing types in Shox2 and Chx10 neuronal populations was statistically significant (${\text{χ}}_{\left(3\right)}^{2}$ = 30.71, *p* < 0.0001). Chx10 neurons preferentially displayed initial doublet firing and Shox2 neurons were mostly tonic firing.

To identify the electrophysiological characteristics that corresponded to the different firing types, we performed statistical analyses on both groups considered as a single population (Shox2 and Chx10 neurons, Figure 4D and Supplementary Table 2). Neurons with delayed firing had lower input resistances than initial doublet firing neurons, longer action potential durations than tonic neurons, shorter slow AHP durations than burst firing neurons, and more depolarized PIC activations than tonic and initial doublet neurons. Initial burst firing neurons had longer slow AHPs and more hyperpolarized PIC activation thresholds than both delay and tonic firing neurons. Initial doublet neurons had longer slow AHPs than tonic firing neurons. Thus, there was no property that distinguished one firing type from all others but there were differences in properties, particularly those related to action potential duration and AHP.

We also compared properties of Shox2 and Chx10 neurons within each firing group (Table 1). Considering only tonic firing neurons, Shox2 neurons had longer time constants (Mann-Whitney test, U = 236, *p* = 0.04), higher capacitance (Mann-Whitney test, U = 199, *p* = 0.01), shorter AP half width (Mann-Whitney test, U = 195.5, *p* = 0.009), and shorter fast (Mann-Whitney test, U = 181, *p* = 0.005) but longer slow (Mann-Whitney test, U = 222, *p* = 0.02) AHPs. We did not find differences between Shox2 and Chx10 neurons with initial doublet firing patterns. The numbers of delayed and initial burst firing Chx10 neurons were low and precluded from statistical analysis. The electrophysiological properties between neurons classified by type of firing were expected to be different since action potential features and spike frequency rely on these properties. Although firing types were differentially distributed in Shox2 and Chx10 populations, it is not possible to separate Shox2 and Chx10 neuronal populations by firing type since the types are common to both.

**Table 1:** Comparison of Shox2 and Chx10 neurons within each firing type.

**Property/Type of cell**	**Tonic**			**Initial doublet**			**Initial burst**		**Delay**	
	**Shox2 (*n* = 78)**	**Chx10 (*n* = 10)**	**p**	**Shox2 (*n* = 36)**	**Chx10 (*n* = 13)**	**p**	**Shox2 (*n* = 21)**	**Chx10 (*n* = 3)**	**Shox2 (*n* = 8)**	**Chx10 (*n* = 2)**
Membrane potential [mV]	−49.7 ± 6	−48.0 ± 6	0.25	−48.6 ± 7	−50.9 ± 6	0.1	−48.5 ± 6	−44.7 ± 3	−50.1 ± 6	−48.0 ± 4
Input resistance [MΩ]	704 ± 363	758 ± 277	0.32	838 ± 433	878 ± 258	0.46	626 ± 241	1,073 ± 795	445 ± 189	841 ± 278
Time constant [ms]	42.5 ± 20	29.8 ± 9	^*^0.04	41.9 ± 22	47.2 ± 12	0.1	37.2 ± 14	37.4 ± 13	41.0 ± 26	43.5 ± 28
Capacitance [pF]	71.1 ± 50	43.1 ± 16	^*^0.01	58.1 ± 33	56.9 ± 17	0.5	66.3 ± 37	42.2 ± 14	108.0 ± 79	60.7 ± 53
Rheobase [pA]	23.3 ± 14	26.9 ± 17	0.4	21.6 ± 11	16.5 ± 7	0.09	24.0 ± 10	18.0 ± 13	28.5 ± 15	22.0 ± 8
AP Threshold [mV]	−36.4 ± 4	−36.5 ± 5	0.4	−35.9 ± 4	−35.6 ± 3	0.7	−37.2 ± 6	−37.7 ± 5	−33.6 ± 4	−32.5 ± 7
AP Half width [ms]	1.01 ± 0.3	1.17 ± 0.1	^*^0.009	1.08 ± 0.2	1.25 ± 0.3	0.05	1.20 ± 0.4	1.12 ± 0.3	1.41 ± 0.4	1.43 ± 0.4
fAHP duration [ms]	4.4 ± 1.8	6.0 ± 2.0	^*^0.005	4.3 ± 1.5	4.7 ± 1.6	0.5	5.3 ± 2.8	7.5 ± 6.2	5.5 ± 1.4	5.9 ± 1.1
sAHP duration [ms]	54.8 ± 34	32.8 ± 26	^*^0.02	101.4 ± 56	80.6 ± 37	0.2	156.1 ± 85	122.4 ± 119	84.1 ± 97	69.0 ± 14
fAHP amplitude	0.3 ± 0.08	0.25 ± 0.07	0.05	0.26 ± 0.08	0.26 ± 0.07	0.6	0.24 ± 0.1	0.29 ± 0.03	0.28 ± 0.06	0.28 ± 0.09
PIC on [mV]	−47.2 ± 5	−49.4 ± 3	0.15	−48.7 ± 4	−50.5 ± 5	0.2	−50.6 ± 6	−54.7 ± 2	−43.6 ± 3	−45.9 ± 7
AP F/I slope	0.36 ± 0.21	0.26 ± 0.15	0.15	0.29 ± 0.17	0.27 ± 0.09	0.9	0.24 ± 0.15	0.11 ± 0.14	0.21 ± 0.18	0.16 ± 0.18

* p < 0.05, t-test or Mann-Whitney comparing Shox2 vs. Chx10.

### k-means cluster analysis to classify Shox2 neuronal subtypes

**Figure 5:**
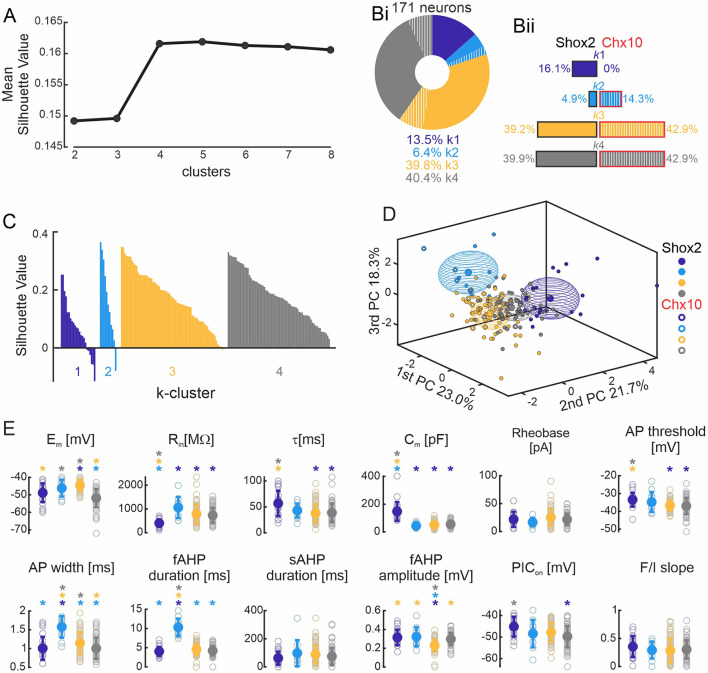
*k*-means clustering. **(A)** Plot of the highest silhouette values for each number of clusters (k, x axis). **(Bi)** Incidence of the four k clusters in 171 neurons, with 83.6% of the neurons from the Shox2 population (solid) and 16.4% were Chx10 neurons (hatched). k1 cluster (purple, *n* = 23), k2 cluster (cyan, *n* = 11), k3 cluster (yellow, *n* = 68), and k4 cluster (gray, *n* = 69). The same color code is used throughout the figure. **(Bii)** Incidence of k clusters observed in 143 Shox2 neurons (left): k1 cluster (*n* = 23), k2 cluster (*n* = 7), k3 cluster (*n* = 56) and k4 cluster (*n* = 57). Incidence of the k clusters observed in 28 Chx10 neurons (right): k1 (*n* = 0), k2 cluster (*n* = 4), k3 cluster (*n* = 12), and k4 cluster (*n* = 12). **(C)** Plot of the cluster silhouette values to demonstrate how similar the individual neurons are to the neurons of their own clusters. Each bar represents a single neuron. **(D)** Three-dimensional representation of all neurons (Shox2 filled dots, Chx10 unfilled dots) on the three principal components (same as Figure 2B but different angle displayed). Ellipsoids are centered on the mean for the three first PC and semi-axes are generated by the standard deviation of each of the three first PCs. **(E)** Comparisons of electrophysiological properties of the neurons from k1, k2, k3 and k4 clusters. Filled circle represents the mean and error bars the standard deviation, unfilled gray circles represent individual neurons. *p < 0.05, one-way ANOVA with Tukey *post-hoc* test or Kruskal-Wallis with Dunn's *post-hoc* test. * significantly different from the corresponding color coded group.

Firing type classification is based on a subjective analysis which could bias the results. To classify Shox2 neurons based on active and passive cellular properties in an unbiased way, we performed a *k*-means cluster analysis (see Methods) on the set of 171 neurons, which included mainly Shox2 neurons and a small sampling of Chx10 neurons to serve as comparison with a known subdivision with the Shox2 population. The cluster analysis considered the six electrophysiological parameters that were not highly correlated, as described above (Figure 2A). To select the number of clusters (k) that best fit the data, we ran the *k*-means algorithm one thousand times from k = 2–8 and plotted the highest silhouette value in each condition (Figure 5A). We found that that the silhouette value reached a plateau from k = 4 to k = 8 and, therefore, we selected k=4 for the analysis.

The *k*-mean cluster algorithm generated four clusters (Figure 5Bi). Two of the clusters contained the majority of the neurons (k3 and k4), with each containing ~40% of the population. Since Shox2 neurons were 83% of the neurons included in the analysis, the distribution of the 143 Shox2 neurons was largely similar to that of the entire population (Figure 5Bii, left side) with 23, 7, 56, and 57 neurons in clusters were k1, k2, k3, and k4, respectively. Of the 28 Chx10 neurons (Figure 5Bii, right side), no neurons were in k1, 4 were in k2, and k3 and k4 each included 12 Chx10 neurons. The difference in the distribution of the 4 k-clusters was statistically significant comparing Shox2 and Chx10 populations (${\text{χ}}_{\left(3\right)}^{2}$ = 22.85, p<0.0001). k1 cluster is composed exclusively of Shox2 neurons (binomial test *p* = 0.01). Chx10 neurons appear more prevalent in k2 cluster than Shox2 neurons, with 14% of Chx10 neurons but only <5% of Shox2 neurons belonging to that cluster, but this is not significant (binomial test *p* = 0.09). The proportion of Chx10 neurons in each of the k3 and k4 clusters (39.2% and 39.9%) is similar to that of Shox2 neurons (42.9% each, binomial test, *p* = 0.7 and *p* = 0.8, for k3 and k4, respectively). The silhouette values for each of the 171 neurons in the k-clusters (Figure 5C) show that most of the neurons have positive values, indicating that the neuron is close to its cluster centroid and far from the centroids of the other clusters. Few neurons (*n* = 8) have negative values which indicate that the neuron is close to its cluster centroid but also close to other cluster centroids. Together, this shows that the k-means analysis (Figure 5D) associates the majority of neurons suitably and that k3 and k4 are comprised of Shox2 and Chx10 neurons rather equally and k1 is exclusively constituted of Shox2 neurons.

To identify the electrophysiological characteristics of each k-cluster, we performed statistical analysis on the 4 k-clusters considering the 171 neurons (Figure 5, Supplementary Table 3). Unlike when separated by firing type, here, there were more differences between clusters in both the 6 properties used for the analysis and the 6 other properties that were found to be highly correlated (Figure 5E, compared to Figure 4D). Neurons in the k1 cluster are distinguished from all other clusters in that they have the lowest input resistance and highest capacitance, suggesting that these are larger neurons. They also had the longest time constant and most depolarized voltage thresholds, although these were not significantly different from the neurons in the k2 cluster. Neurons in the k2 cluster can be distinguished from the other clusters by their longer AP and fast AHP durations. Neurons in the k3 cluster have the largest fast AHPs and have the most depolarized membrane potentials, although the latter is not different than neurons in the k2 cluster. Neurons in the k4 cluster have the most hyperpolarized resting membrane potentials and narrow action potential, although neither of these are significantly different from k1 neurons. Neurons in the k4 cluster have more hyperpolarized PIC activation voltages than neurons in k1. Taken together, this suggests that k1 cluster properties may be characteristic of Shox2^+^Chx10^−^ neurons while properties of k3 and k4 clusters, and k2 cluster to a lesser extent, may be characteristic of Shox2^+^Chx10^+^ neurons.

We next performed statistical analysis of the k clusters considering Shox2 and Chx10 neurons separately (Table 2). In k2, which had a higher proportion of Chx10 neurons and few Shox2 neurons, the only significant difference was that C_m_ of k2 Shox2 neurons was higher than of k2 Chx10 neurons (Mann-Whitney test, U = 2, p = 0.02). We did not find differences in k3 cluster (with equal proportions of Shox2 and Chx10 neurons) between Shox2 and Chx10 populations. The k4 also had equal proportions of Shox2 and Chx10 neurons and the only differences between the Shox2 and Chx10 neurons in that cluster were in AP halfwidth (Mann-Whitney test, U = 168.5, *p* = 0.005) and fast AHP amplitude (unpaired *t* test, *t*_(2.04)_ = 67, *p* = 0.04). Notably, there were less differences between Shox2 and Chx10 neurons from individual k clusters than there were differences in the whole populations of Shox2 and Chx10 neurons. This demonstrates similarity within clusters regardless of the transcription factor expressed.

**Table 2:** Comparison of properties of Shox2 and Chx10 neurons in each k cluster.

**Property/Cluster**	**k1**	**k2**			**k3**			**k4**		
	**Shox2 (*n* = 23)**	**Shox2 (*n* = 7)**	**Chx10 (*n* = 4)**	**p**	**Shox2 (*n* = 56)**	**Chx10 (*n* = 12)**	**p**	**Shox2 (*n* = 57)**	**Chx10 (*n* = 12)**	**p**
Membrane potential [mV]	−48.8 ± 5	−47.3 ± 5	−44.3 ± 3	0.4	−44.4 ± 3	−45.3 ± 4	0.4	−51.7 ± 5	−52.3 ± 5	0.4
Input resistance [MΩ]	408 ± 160	993 ± 388	1,190 ± 567	0.9	797 ± 407	770 ± 301	0.8	716 ± 321	825 ± 226	0.3
Time constant [ms]	56.9 ± 24	47.9 ± 11	35.5 ± 14	0.2	37.9 ± 18	37.9 ± 14	0.7	37.9 ± 18	43.1 ± 15	0.2
Capacitance [pF]	148.3 ± 67	53.1 ± 16	30.4 ± 8	^*^0.02	51.6 ± 20	52.9 ± 19	0.9	56.5 ± 21	55.1 ± 20	0.9
Rheobase [pA]	21.9 ± 14	16.9 ± 4	16.8 ± 12	0.9	25.1 ± 15	25.8 ± 15	0.8	22.9 ± 11	17.0 ± 8	0.07
AP threshold [mV]	−33.4 ± 4	−32.6 ± 6	−38.5 ± 2	0.4	−36.6 ± 2	−36.7 ± 5	0.9	−37.5 ± 5	−34.3 ± 4	0.5
AP half width [ms]	1.01 ± 0.3	1.71 ± 0.2	1.35 ± 0.4	0.2	1.14 ± 0.3	1.20 ± 0.2	0.5	1.00 ± 0.3	1.20 ± 0.3	^*^0.005
fAHP duration [ms]	4.1 ± 1.2	10.2 ± 2.0	10.5 ± 3.1	0.9	4.5 ± 1.7	4.8 ± 1.0	0.5	4.1 ± 1.3	4.7 ± 1.1	0.1
sAHP duration [ms]	62.9 ± 46	120.1 ± 107	60.0 ± 48	0.3	93.6 ± 64	72.2 ± 63	0.2	76.3 ± 64	76.9 ± 42	0.5
fAHP amplitude	0.32 ± 0.08	0.32 ± 0.13	0.32 ± 0.04	0.8	0.23 ± 0.07	0.24 ± 0.05	0.5	0.31 ± 0.06	0.26 ± 0.07	^*^0.04
PIC on [mV]	−45.2 ± 5	−46.4 ± 7	−52.1 ± 3	0.07	−47.5 ± 4	−49.0 ± 6	0.3	−49.5 ± 5	−50.8 ± 4	0.4
AP F/I slope	0.36 ± 0.19	0.32 ± 0.12	0.24 ± 0.21	0.5	0.31 ± 0.23	0.19 ± 0.11	0.07	0.31 ± 0.17	0.29 ± 0.10	0.7

* p < 0.05, t-test or Mann-Whitney comparing Shox2 vs. Chx10.

### Hierarchical cluster analysis to classify subpopulations of Shox2 and Chx10 neurons

**Figure 6:**
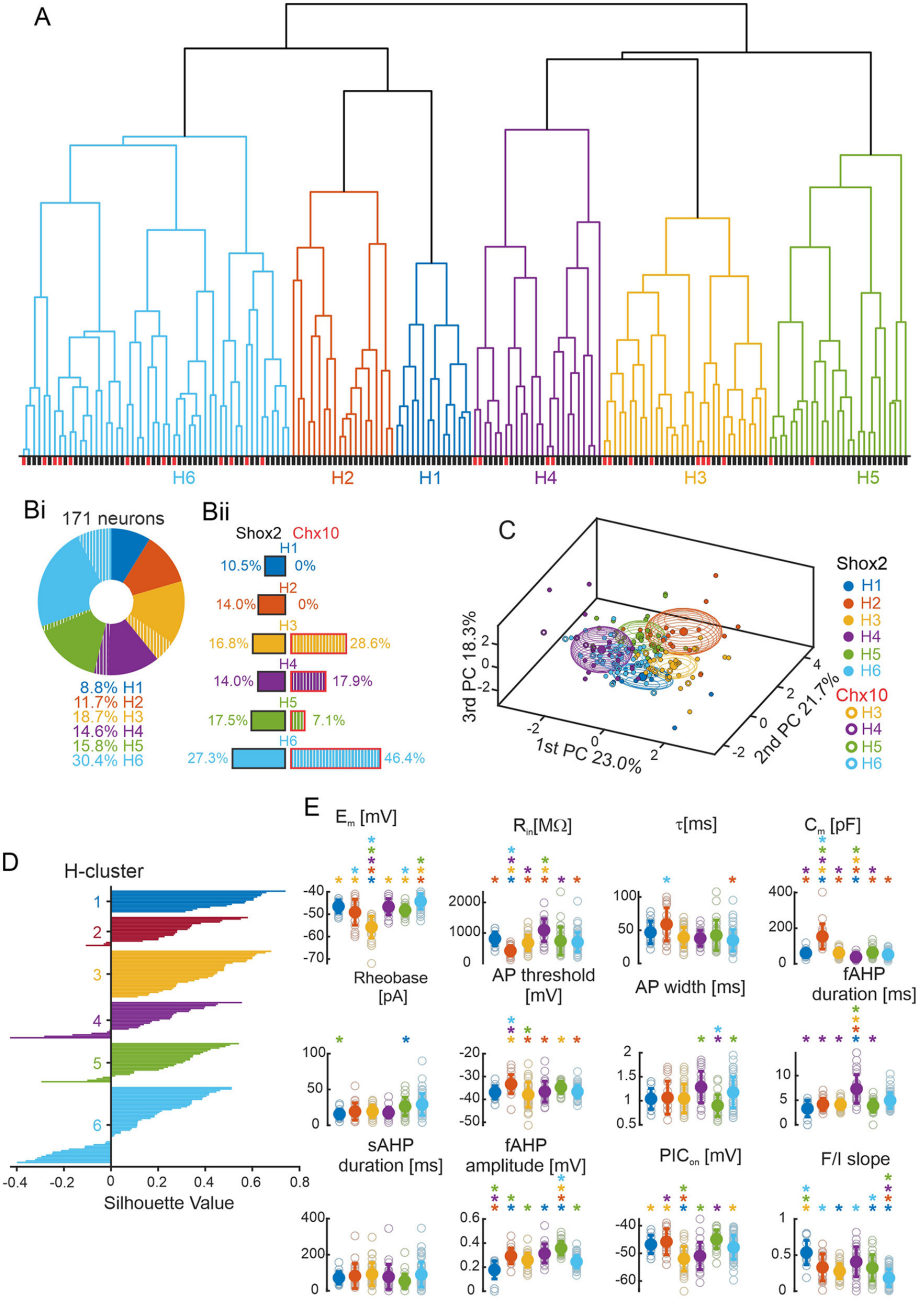
Hierarchical clustering. **(A)** Dendrogram plot of the hierarchical binary cluster tree of 171 neurons. Clusters are generated by natural divisions with the cutoff below the value of the maximum inconsistency coefficient. Clusters are color coded: blue for H1 cluster, orange for H2 cluster, yellow for H3 cluster, purple for H4 cluster, green for H5 cluster, and cyan for H6 cluster. The same color code is used for the other panels. The lines below correspond to the type of neurons with Shox2^+^ neurons in black and Chx10^+^ neurons in red. **(Bi)** Incidence of the six H clusters in all 171 Shox2 neurons (solid) and Chx10 neurons (hatched). H1 cluster (*n* = 15), H2 cluster (*n* = 20), H3 cluster (*n* = 32), H4 cluster (*n* = 25), H5 cluster (*n* = 27) and H6 cluster (*n* = 52). **(Bii)** Incidence of the H clusters observed in 143 Shox2 neurons (left): H1 (*n* = 15), H2 (*n* = 20), H3 (*n* = 24), H4 (*n* = 20), H5 (*n* = 25) and H6 cluster (*n* = 39). Incidence of the H clusters observed in 28 Chx10 neurons (right): H1 and H2 (*n* = 0), H3 (*n* = 8), H4 (*n* = 5), H5 (*n* = 2) and H6 cluster (*n* = 13). **(C)** Three-dimensional representation of all neurons (Shox2 filled, Chx10 open) on the three principal components (same as Figure 2B but a different angle). Ellipsoids are centered on the mean for the three first PCs and semi-axes are generated by the standard deviation of each three first PC. **(D)** Plot of the cluster silhouettes values. **(E**) Comparisons of electrophysiological properties of the neurons from H1, H2, H3, H4, H5 and H6 clusters. Filled circles and error bars represent the mean and standard deviation, open gray circles represent individual neurons. *p < 0.05, one-way ANOVA with Tukey *post-hoc* test or Kruskal-Wallis with Dunn's *post-hoc* test. *significantly different from the corresponding color coded group.

The number of clusters in the k-means analysis here was defined by the elbow rule on a graph of the silhouette values (Allen et al., 2014; Vergara et al., 2020) which can be considered arbitrary. Hierarchical clustering produces a dendrogram that facilitates the visualization of natural clustering, and, therefore, represents a more unbiased method of classification (Martinez et al., 2017; Di Miceli et al., 2020). Thus, we performed an unbiased hierarchical clustering (see Methods) considering the same set of data used for k-means algorithm (171 neurons, 6 variables in Figure 2Aii). The algorithm established 6 clusters generated by natural divisions with cutoffs below the value of the maximum inconsistency coefficient. The component neurons of each hierarchical cluster (H cluster) can be visualized in a dendrogram (Figure 6A).

Each of the six H clusters (Figure 6Bi) contained 8.8–30.4% of the input population. Based on the silhouette values and the ellipsoids on the PCA graphs (Figures 6C,D), the first 5 clusters described their constituent neurons well with mainly positive values, where the largest cluster was H6 and 29% of neurons had negative values. When considering the 143 Shox2 neurons (Figure 6Bii, left) separately, neurons were relatively evenly distributed between H1–H5 (*n* = 15–25 in each) with more neurons in H6 (*n* = 39). The 28 Chx10 neurons (Figure 6Bii, right) were distributed between H3–H6 clusters (*n* = 2–13) but there are no Chx10 neurons in H1 and H2 clusters. The difference in the distribution of the 6 H-clusters was statistically significant when comparing Shox2 and Chx10 populations (${\text{χ}}_{\left(5\right)}^{2}$ = 14.95, *p* = 0.01). Note that H1 and H2 clusters were composed of Shox2 neurons exclusively and these two clusters were most similar to each other, as seen by the height of the next branch point in the analysis. Although clusters H3-H6 contained both Shox2 and Chx10 neurons, the uneven numbers of Shox2 and Chx10 input neurons (143 vs. 28) should be considered (Figure 6Bii). H3 and H6 clusters were enriched in Chx10 neurons, H5 was enriched in Shox2 neurons, and the proportions of Shox2 and Chx10 neurons in H4 were similar.

To identify the electrophysiological characteristics of each H-cluster, we performed statistical analysis on these clusters considering all 171 neurons (Figure 6E, Supplementary Table 4). Neurons in the H1 cluster displayed the smallest fast AHP amplitudes and highest firing frequencies in response to depolarizing current steps but these were not significantly different from all other clusters. The neurons in the H2 cluster had the lowest input resistance (significantly different from all but H5), highest capacitance, and most depolarized AP threshold (significantly different from all but H1 and H5). Neurons in the H3 cluster had the most hyperpolarized resting membrane potentials, AP thresholds more hyperpolarized than neurons in H2 and H5, and the most hyperpolarized PIC activations (significantly different from all but H4). Neurons in the H4 cluster had the highest input resistances, lowest capacitance values, and longest fast AHP durations, although none of these properties were significantly different from neurons in the H6 cluster. Neurons in the H5 cluster had the highest fast AHP amplitude (significantly different from all but H4). Lastly, neurons in the H6 cluster had the lowest firing frequencies in response to depolarizing current steps (F-I slope, significantly different from all but H3). Thus, hierarchical clustering generated six distinct clusters, two of which were composed only of Shox2 neurons, suggesting that larger neurons (low R_in_, high C_m_) with depolarized voltage thresholds are characteristic of Shox2^+^Chx10^−^ neurons.

We also performed statistical analysis of the H clusters considering Shox2 and Chx10 neurons separately (Table 3). There were no Chx10 neurons in H1 and H2 clusters for analysis and H5 contained only 2 Chx10 neurons which precluded statistical testing. There were no differences between Shox2 and Chx10 neurons in the H6 cluster. There were only a few differences between Shox2 and Chx10 neurons in H3 and H4 clusters. Shox2 neurons in H3 had more depolarized AP thresholds and shorter AP half widths than Chx10 neurons in H3. In H4, the only significant difference was that Shox2 neurons had higher capacitance than the Chx10 neurons. Similar to the *k*-means clusters, there were few differences between Shox2 and Chx10 neurons within any of the hierarchical clusters. These were outnumbered by differences between the clusters, suggesting relatively homogeneous populations within each cluster.

**Table 3:** Comparison of properties of Shox2 and Chx10 neurons in each of the hierarchical clusters.

**Property/Cluster**	**H3**			**H4**			**H5**			**H6**		
	**Shox2 (*n* = 24)**	**Chx10 (*n* = 8)**	**p**	**Shox2 (*n* = 20)**	**Chx10 (*n* = 5)**	**p**	**Shox2 (*n* = 25)**	**Chx10 (*n* = 2)**	**p**	**Shox2 (*n* = 39)**	**Chx10 (*n* = 13)**	**p**
Membrane potential [mV]	−56.0 ± 5	−55.0 ± 2	0.9	−46.9 ± 4	−45.8 ± 1	0.5	−48.3 ± 3	−48.0 ± 1	N/A	−44.1 ± 3	−44.9 ± 4	0.5
Input resistance [MΩ]	640 ± 266	781 ± 202	0.2	1,074 ± 348	1,162 ± 495	0.9	731 ± 475	801 ± 423	N/A	673 ± 328	788 ± 296	0.1
Time constant [ms]	36.4 ± 15	47.9 ± 14	0.06	39.8 ± 11	30.0 ± 12	0.07	42.6 ± 24	39.9 ± 14	N/A	33.6 ± 17	38.2 ± 13	0.2
Capacitance [pF]	60.8 ± 23	63.0 ± 17	0.8	40.8 ± 15	26.5 ± 3	^*^0.01	64.6 ± 27	52.4 ± 10	N/A	52.0 ± 16	52.1 ± 18	0.8
Rheobase [pA]	20.6 ± 9	16.0 ± 8	0.2	18.1 ± 7	16.0 ± 10	0.5	26.8 ± 13	20.5 ± 15	N/A	30.4 ± 16	25.5 ± 15	0.3
AP threshold [mV]	−39.3 ± 5	−34.0 ± 5	^*^0.02	−36.2 ± 5	−38.1 ± 2	0.6	−34.9 ± 3	−32.9 ± 2	N/A	−36.6 ± 2	−36.7 ± 4	0.9
AP half width [ms]	0.98 ± 0.3	1.24 ± 0.3	^*^0.03	1.31 ± 0.4	1.20 ± 0.1	0.7	0.89 ± 0.2	1.00 ± 0.4	N/A	1.15 ± 0.3	1.25 ± 0.3	0.4
fAHP duration [ms]	3.9 ± 1.0	4.6 ± 0.9	0.1	6.9 ± 2.6	8.9 ± 3.8	0.3	3.9 ± 1.3	3.8 ± 1.5	N/A	4.9 ± 1.7	5.1 ± 1.5	0.4
sAHP duration [ms]	94.4 ± 70	85.3 ± 46	>0.9	86.1 ± 73	42.3 ± 46	0.1	53.7 ± 42	52.3 ± 24	N/A	97.9 ± 72	67.8 ± 61	0.2
fAHP amplitude	0.27 ± 0.06	0.24 ± 0.07	0.6	0.31 ± 0.09	0.32 ± 0.04	0.6	0.36 ± 0.06	0.32 ± 0.04	N/A	0.25 ± 0.05	0.27 ± 0.05	>0.9
PIC on [mV]	−51.9 ± 5	−52.1 ± 4	0.9	−50.6 ± 6	−52.5 ± 2	0.5	−44.9 ± 4	−44.8 ± 2	N/A	−47.5 ± 4	−49.0 ± 6	0.3
AP F/I slope	0.28 ± 0.02	0.28 ± 0.03	0.8	0.44 ± 0.20	0.29 ± 0.21	0.2	0.33 ± 0.19	0.24 ± 0.17	N/A	0.18 ± 0.13	0.2 ± 0.11	0.7

* p < 0.05, t-test or Mann-Whitney comparing Shox2 vs. Chx10.

### Comparison of k-mean and hierarchical clusters

**Figure 7:**
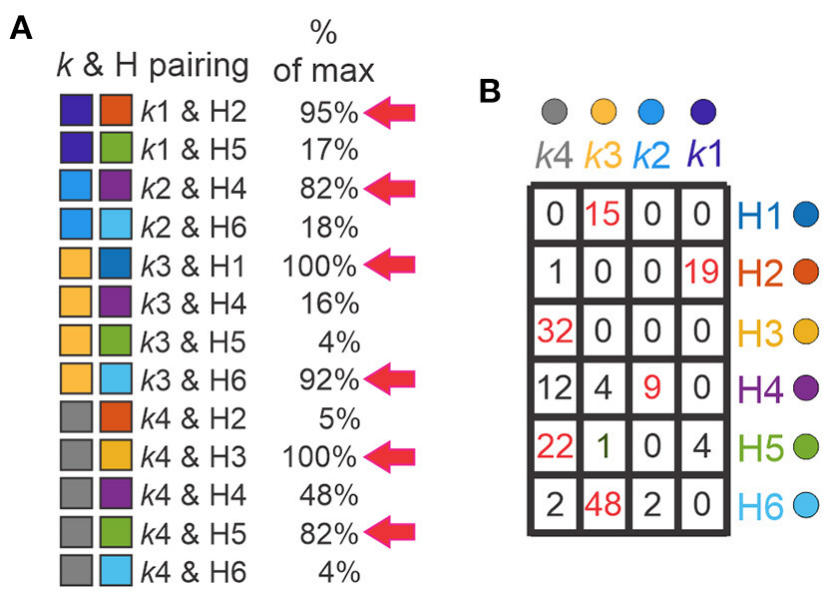
Comparison between k and H clusters. **(A)** Thirteen possible combinations between *k*-cluster and H-clusters (k & H pairing, left) with the corresponding maximum percentage of appearance (right). The pairings with percentages higher than 80% are marked with red arrows. *k*-clusters (left boxes), k1 (purple), *k*2 (blue), *k*3 (yellow), *k*4 (gray), H-clusters (right boxes), H1 (blue), H2 (orange), H3 (yellow), H4 (purple), H5 (green) and H6 (cyan). **(B)** Table shows the k & H pairing. Numbers indicate the neurons within the corresponding cluster, k in columns and H in rows. Red numbers are the neurons with pairing with percentages higher than 80%.

In order to validate clustering results and determine the differences between them, we paired the clusters generated by *k*-means and hierarchical algorithms (Supplementary Figure 1). *k*-means and H clusters overlapped in 13 combinations (Figure 7A, left) and showed high correspondence. Considering the H clusters with respect to the k clusters, all 15 of the neurons in H1 corresponded to k3, all (32) in H3 were in k4, and nearly all (19 of 20) of the neurons in H2 were in k1. Further, the majority of H5 and H6 neurons were in k4 and k3, respectively, where neurons in H4 were distributed but absent from k1. The correspondence can also be considered the other way around. Most neurons in the k1 cluster (19 of 23) are in H2, most neurons in k2 (9 of 11) correspond to H4 neurons, neurons belonging to k3 were mainly distributed on H1 and H6, and neurons in k4 neurons were dispersed but mainly in H3 and H5 (Figure 7B). Furthermore, there were 6 combinations with high overlap (>80% of maximum possibilities of pair appearance, Figure 7A, right). These 6 combinations accounted for 86% of the 171 input neurons (143 Shox2 neurons and 25 Chx10 neurons).

### Further validation of clustering with immunohistochemistry identified cells

**Figure 8:**
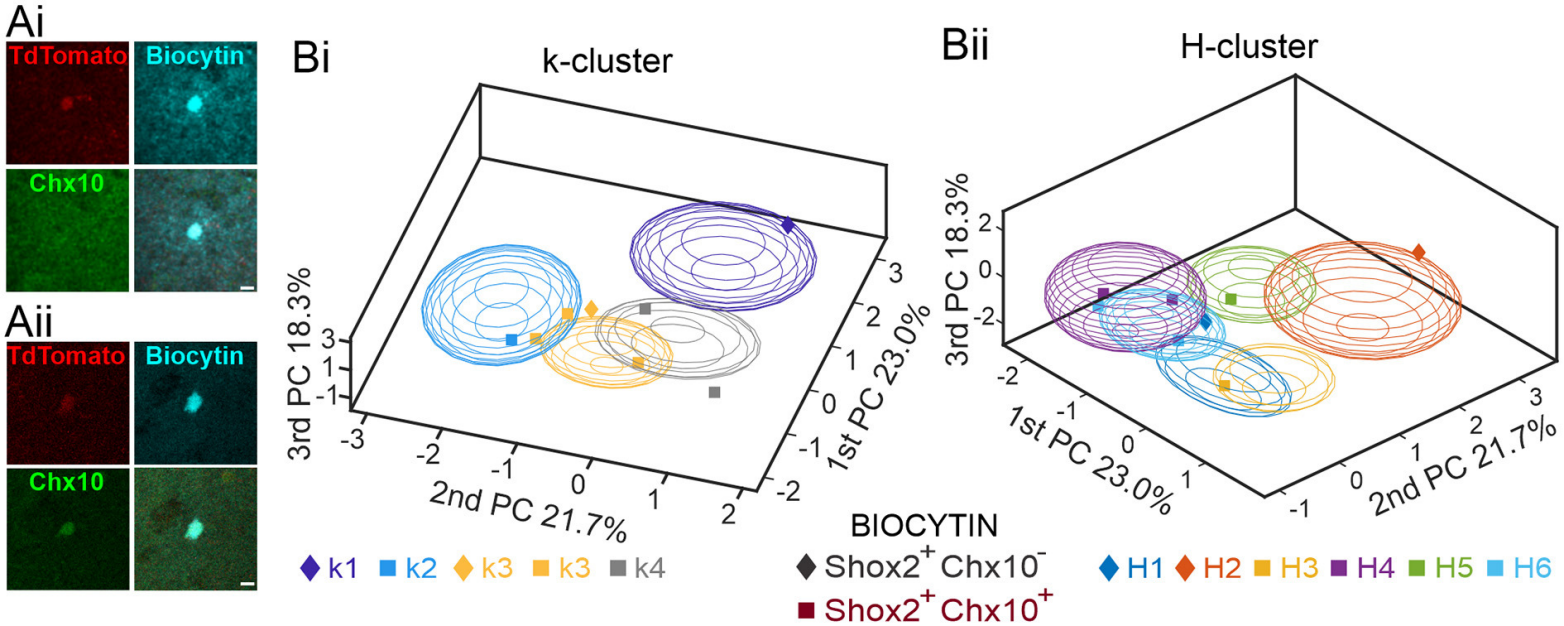
Validation of clustering analyses with Chx10 staining of Shox2 neurons. **(A)** Examples of a Shox2^+^Chx10^−^ **(Ai)** and a Shox2^+^Chx10^+^ **(Aii)** neuron filled with biocytin (cyan) during recording of an identified Shox2 neuron (tdTomato, red) and stained with a Chx10 antibody. **(B)** Three-dimensional representations of 8 Shox2 neurons processed for Chx10 identity (Shox2^+^Chx10^−^, diamonds; Shox2^+^Chx10^+^, squares) on the three principal components (same as Figure 2B, but different angle) for k-mean clusters **(Bi)** and hierarchical clustering **(Bii)**. Ellipsoids are centered on the mean for the three first PC and sem-axes are generated by the standard deviation of each of the three first PCs.

A subset of the Shox2 neurons used for this analysis were recorded with biocytin in the electrode and were recovered for labeling with an antibody to Chx10. In total, we stained 8 neurons to determine Shox2^+^Chx10^+^ or Shox2^+^Chx10^−^ identity (Figure 8A). Of those stained, 2 Shox2 neurons were Chx10^−^ (Shox2^+^Chx10^−^) and 6 Shox2 neurons expressed Chx10 (Shox2^+^Chx10^+^). We next determined which clusters each of the 8 neurons belonged to. Considering k clusters (Figure 8Bi), we found that Shox2^+^Chx10^−^ were classified in k1 (1 neuron) and k3 (1 neuron) clusters, while Shox2^+^Chx10^+^ neurons were classified in k2 (1 neuron), k3 (2 neurons), and k4 (1 neuron) clusters. This matches rather well with predictions from the clustering results (Figure 5) because k1 contained only Shox2 neurons and k2–k4 contained both Shox2 and Chx10 neurons. Considering H clusters (Figure 8Bii), we found that Shox2^+^Chx10^−^ where classified in H1 (1 neuron) and H2 (1 neuron), the clusters devoid of Chx10 neurons. The Shox2^+^Chx10^+^ neurons were in H3 (1 neuron), H4 (2 neurons), H5 (1 neuron) and H6 (2 neurons), all clusters which contain both Shox2 and Chx10 neurons. Thus, these results further support the ability of the H clustering method to distinguish populations of Shox2 neurons.

## Discussion

In the present study, electrophysiological properties were used to define populations of adult spinal Shox2 interneurons. Since Shox2 neurons overlap partly with Chx10-expressing neurons (Dougherty et al., 2013), we included a group of Chx10 neurons in our analysis. We classified the neurons based on their type of firing into 4 groups, and there were few significant differences between the groups in terms of electrophysiological properties. On the contrary, computational clustering methods delineated groups which could be readily distinguished by active and passive membrane properties. The *k*-means algorithm was run for 4 populations of neurons while hierarchical clustering analysis defined 6 populations. Interestingly, in each of the two clustering analyses, we found clusters containing exclusively Shox2 neurons, suggesting possible definition of Shox2^+^Chx10^−^ populations by electrophysiological properties. Finally, as preliminary validation, Chx10 antibody staining of a small subset of biocytin-filled and recovered Shox2 neurons showed that all (8/8) of the filled and post-processed neurons were appropriately found in the expected hierarchical (H) clusters based on Chx10 presence/absence. The *k*-means clustering corresponded to the expected in 7/8 cases. Taken together, the data demonstrate that it is possible to classify neurons expressing Shox2 in at least six different subpopulations based on active and passive membrane properties. These subpopulations correspond well with Chx10 absence/presence and may provide further divisions between neurons currently defined with intersectional genetics.

### Classification by firing type

The firing type of neurons, defined by the response to current steps, is used to classify interneurons throughout the CNS (Lopez-Garcia and King, 1994; Prescott and De Koninck, 2002; Ruscheweyh and Sandkuhler, 2002; Dougherty and Hochman, 2008; Dai et al., 2009; Dougherty and Kiehn, 2010; Yasaka et al., 2010; Zhong et al., 2010; Dougherty and Chen, 2016). Prior studies have noted that Shox2 and Chx10 neurons are not entirely homogeneous (Dougherty and Kiehn, 2010; Zhong et al., 2010; Dougherty et al., 2013; Ampatzis et al., 2014; Garcia-Ramirez et al., 2021). There were significant differences in the distribution of neurons classified based on the type of firing. The majority of Shox2 neurons displayed a tonic response while the majority of Chx10 neurons displayed initial doublet firing. Adult Chx10 neurons have been shown to be predominantly tonic firing (Husch et al., 2015) which aligns with 83% of the Chx10 neurons in the present study firing throughout the current step. However, over half of the Chx10 neurons firing throughout a current step here had an initial doublet. The discrepancy may be due to the level of description, type of recording (whole-cell vs. perforated patch), or differences in neurons maintaining fluorescence expression in the mouse lines used (Chx10-CFP vs Chx10GFP). Initial burst (called phasic in some studies) and delayed firing types have been reported in neonatal Chx10 neurons (Dougherty and Kiehn, 2010; Zhong et al., 2010). The types of firing patterns observed are largely consistent between neonatal and adult but shifts in the distributions with maturation have been noted previously (Husch et al., 2015).

### Unbiased cluster analysis to classify Shox2 and Chx10 neurons

The differences in the groups generated based on the type of firing was low. Additionally, a possible subclassification that merged Shox2 and Chx10 populations was not evident. Furthermore, subjectivity is another disadvantage for this type of classification. Unsupervised computational algorithms, have been used to unbiasedly classify neuronal populations based on physiological, molecular, and anatomical features (Dombeck et al., 2009; Karagiannis et al., 2009; Helm et al., 2013; Li et al., 2017; Martinez et al., 2017; Sathyamurthy et al., 2018; Mickelsen et al., 2019; Di Miceli et al., 2020). Two of the most prevalent algorithms implemented in such analyses are k-means and hierarchical clustering (Armananzas and Ascoli, 2015; Zeng and Sanes, 2017). Both analyses group individual objects (in this case neurons) based on similarities in input data (i.e. electrophysiological characteristics). The k-means algorithm requires a pre-established number of clusters (k value) set by the operator. We tested the k-mean algorithm in a range from 2 to 8 clusters, based on the idea of at least two populations of Shox2 neurons depending on the expression of Chx10 (Dougherty et al., 2013) and at least four groups based on the type of firing. We chose to proceed with 4 clusters because that resulted in the highest mean silhouette value (Allen et al., 2014; Vergara et al., 2020). However, we do not exclude the possibility of finding higher silhouette values with more than 8 clusters. The hierarchical cluster algorithm does not require the specification of the number of clusters prior to initial analysis. We found 6 natural divisions in the data based on a cutoff threshold below the value of the maximum inconsistency. Although the analyses resulted in a different number of clusters there was a high overlap between the two methods when clusters were directly compared.

A main motivation for the analyses was to determine if there was a way to identify subpopulations within a class of interneurons using electrophysiological properties rather than combinatorial genetics. The neurons expressing Shox2 can be divided into 2 subpopulations based on the expression of Chx10, Shox2^+^Chx10^−^ and Shox2^+^Chx10^+^. When considering both transcription factors, it should be noted that there are also Chx10 neurons which do not express Shox2 (Shox2^−^Chx10^+^). One may expect that electrophysiological properties are related to transcription factor expression and inferred function. Thus, there would be clusters that contain each of the possible combinations. If this is the case, the distribution of Shox2 and Chx10 neurons in the clusters identified by the *k*-means and hierarchical algorithms could potentially be used to predict the type of neurons (Shox2^+^Chx10^−^ or Shox2^+^Chx10^+^) that correspond to each cluster. For example, the lack of Chx10 neurons in k1, H1 and H2 clusters suggest that these clusters are composed of Shox2^+^Chx10^−^ neurons. H5 cluster could also be considered as a putative Shox2^+^Chx10^−^ cluster, since 93% of H5 cluster is comprised by Shox2 neurons, suggesting higher influence of Shox2 features than Chx10. As k3, k4, H3, H4 and H6 clusters have similar distributions of Shox2 neurons (82, 83, 75, 80 and 75%, respectively) as the total proportion of Shox2 neurons sampled (83%), we suggest that these clusters are composed of Shox2^+^Chx10^+^ neurons. In contrast, Shox2 neurons constitute 64% of the neurons in k2. Even though this percentage is lower than the total distribution of Shox2, this is not significantly different (binomial test, *p* = 0.09) and therefore we cannot classify them as Shox2^−^Chx10^+^ neurons.

We do not have any clusters of Shox2^−^Chx10^+^ neurons. This is most likely because there is a low number of Chx10 neurons in our analysis which resulted in an underrepresentation of the population of Shox2^−^Chx10^+^ neurons. Our primary focus here was the division of the Shox2 population and how it would match up with the subgroupings by transcription factor (Chx10) expression. A future analysis may include a more balanced sample of Chx10 neurons, and we expect that would generate clusters consisting of only Chx10 neurons.

**Figure 9:**
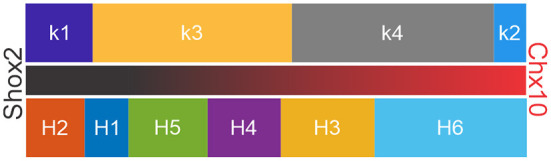
Arrangement of clusters in suggested order of gradient of Shox2-like to Chx10-like properties. k-means (above) and hierarchical (below) clusters arranged based on their similitudes with Shox2 (left and black portion of the middle bar) and Chx10 (right and red portion of the middle bar) neuronal characteristics.

In order to validate clusters obtained from both hierarchical and *k*-mean algorithms, previous studies have compared the output of both clusters and then corrected one or both algorithms (Karagiannis et al., 2009; McGarry et al., 2010; Perrenoud et al., 2012; Helm et al., 2013; Pohlkamp et al., 2014; Martinez et al., 2017). Here, we compared *k*-mean and hierarchical clusters and found that in 86% of the neurons were found in corresponding k-means and H-cluster pairs. Based on the distribution of Shox2 and Chx10 neurons in the k and H clusters, neurons can be arranged in a gradient from most Shox2 neuron-like to a most Chx10 neuron-like (Figure 9). The fact that k1, H1 and H2 clusters are composed only of Shox2 neurons suggests that these clusters have the features of Shox2^+^Chx10^−^ neurons. k3, k4, H3, H4 and H6 are composed of Shox2^+^Chx10^+^ neurons and the k2 cluster has an inclination toward a Chx10 phenotype. The arrangement displayed here could be considered as distinct populations of neurons or a phenotypic gradient of Shox2 to Chx10 neuronal features.

### Electrophysiological characteristics of Shox2 and Chx10 neurons

The passive and active electrophysiological properties of the neurons delineate firing responses of the cell to synaptic inputs (Russo and Hounsgaard, 1999; Butt et al., 2002; Smith and Perrier, 2006; Grillner and El Manira, 2020). Shox2 and Chx10 neurons are involved on the generation of the locomotor rhythm and pattern (Crone et al., 2008; Dougherty and Kiehn, 2010; Zhong et al., 2010, 2011; Dougherty et al., 2013; Dougherty and Ha, 2019). Therefore, the electrophysiological passive and active properties of the neuronal clusters and the differences between them should provide insights into the neuronal characteristics required for unique functional populations. We found that Shox2 neurons have lower input resistances and higher capacitance than Chx10 neurons, suggesting that Shox2 neurons are larger than Chx10 neurons. In fact, k1 and H2 clusters, that we consider to be Shox2^+^Chx10^−^ neurons also displayed low input resistances and high capacitances. On the other hand, the H4 cluster linked with a more Shox2^+^Chx10^+^-like phenotype had high input resistances and low capacitances.

One characteristic of motoneurons is their spike frequency adaptation in response to depolarizing current steps. Spike frequency adaptation is related to the inactivation of Na^+^ channels and functionally related to the initiation of muscle contraction (Miles et al., 2005; Brownstone, 2006). The initial doublet and tonic firing types observed here differed in spike frequency adaptation displayed. Spike frequency adaptation is evident in the low slope values from initial double compared with tonic neurons (Table 2). The significant differences observed in the percentage of type of firing between Shox2 and Chx10 are due to mainly to the larger proportion of Chx10 neurons with the initial doublet firing and higher incidence of the tonic type of firing for Shox2 neurons. Chx10 neurons also displayed longer action potentials and fAHPs in comparison to Shox2 (Table 2). In motoneurons, the fAHP has been linked to the activation of high threshold transient A-type K^+^ channels (Hess and El Manira, 2001; Harris-Warrick, 2002). fAHP contributes to the adaptation observed in initial doublet firing neurons (Baldissera and Gustafsson, 1974; Madison and Nicoll, 1984; Mrowczynski et al., 2015). Here, we found reduced fAHP amplitudes in neurons in the H1 cluster (Shox2^+^Chx10^−^) and longer fAHP durations in neurons in the k2 cluster (Shox2^−^Chx10^+^ enriched), which corresponds well with the proportion of initial doublet firing neurons in each group. Taken together, the computational clustering analysis performed here exposed potential novel differences between Shox2 populations that allow for hypotheses to be made regarding the characteristics of these populations. It is important to note that the electrophysiological recordings were performed at room temperature, as with most recordings of adult locomotor-related neurons *in vitro* (Husch et al., 2012; Mitra and Brownstone, 2012; Hadzipasic et al., 2014; Smith and Brownstone, 2020). The dynamics and activity of ion channels may differ at physiological temperatures (Russell et al., 1994; Li and Burke, 2001; Robertson and Money, 2012; O'Leary and Marder, 2016). Although it is likely to affect values used here for classification, diversity in properties is seen in spinal neurons *in vivo* (Graham et al., 2004) and similar clustering methodologies should be applicable.

### Computational clustering impact

Recent advances in computational technologies have increased our capabilities to cluster spinal neurons, based on molecular profiles from RNA sequence analysis (Li et al., 2017; Menon, 2018; Sathyamurthy et al., 2018; Mickelsen et al., 2019). RNA sequence analysis provides us information about the possibilities of neurons to express proteins that in turn will form ion channels or define neurotransmitter machinery. But, the ways by which these proteins actively participate during behavior is dynamic (Hager et al., 2009; Tanay and Regev, 2017). Classification based on electrophysiological properties, should be considered as a complement to the biomolecular identification of neurons. Clustering methods with electrophysiological properties have been used to identify neurons from other areas of the central nervous system (Karagiannis et al., 2009; Perrenoud et al., 2012; Helm et al., 2013; Martinez et al., 2017; Di Miceli et al., 2020). Here, in addition to computational clustering to classify Shox2 and Chx10 neurons, we were able to test the predicted clusters with immunocytochemistry approaches with high accuracy. The results observed and further validated allow some of the electrophysiological properties that potentially conferred the capacity to a group of neurons to perform specific locomotor task to be inferred.

Limitations of computational clustering techniques include the necessity of a large sample of neurons for the analysis (Armananzas and Ascoli, 2015). Additionally, a large number of parameters should be measured for each neuron in the sample (Armananzas and Ascoli, 2015). Although here we demonstrate that it is possible with a more limited number of parameters, the larger number was collapsed by determining those that were highly correlated. Another limitation is that most clustering algorithms (including the two used here) require the number of clusters generated to be pre-determined. In order to minimize subjectivity in the number of clusters, these can be determined based on mathematical criteria, as we did here. For k-means, the elbow method considering silhouette values can be used (Allen et al., 2014; Vergara et al., 2020) and a cutoff can be established considering inconsistency coefficients for hierarchical clustering (McGarry et al., 2010). However, computational clustering overcomes limitations of other methods by being unbiased. For example, the classification of neurons based on type of firing is subjective and, therefore, susceptible to discrepancies. Both k-means and hierarchical clustering are easy to implement, the computations are fast, and they consider all of the data including apparent outliers (Karagiannis et al., 2009; Armananzas and Ascoli, 2015).

The computational unsupervised clustering technique used here would be useful to classify subpopulations of spinal interneurons in multiple applications. It could be used when recording from neurons in *in vitro* preparations to avoid the necessity of combinatorial transgenic mice. Additionally, *in vivo* recordings often performed blindly (Tao et al., 2015; Lee and Lee, 2017) but more intact preparations are important for the understanding of neuronal spinal physiology in awake animal models. Furthermore, electrophysiological methods to classify neurons could be combined with other types of classifications, such as large scale snRNA-sequencing (Sathyamurthy et al., 2018; Patterson-Cross et al., 2021) or anatomical criteria (Karagiannis et al., 2009; McGarry et al., 2010), to obtain a multidimensional identification of spinal neuronal populations. Finally, the cluster analysis performed here could eventually be converted to a semi-supervised machine learning model by training the algorithm to classify a larger scale of spinal neurons (Buccino et al., 2018; Paninski and Cunningham, 2018) that is capable of considering additional factors including behavioral responses, connectivity, and/or neuromodulation, thereby integrating cellular physiology to behavior.

## Data availability statement

The original contributions presented in the study are included in the article/Supplementary material, further inquiries can be directed to the corresponding author.

## Ethics statement

The animal study was reviewed and approved by Institutional Animal Care and Use Committee at Drexel University.

## Author contributions

DG-R and KD designed the study, drafted the manuscript, and wrote the manuscript. DG-R, SS, JM, and NH performed the electrophysiological recordings and individual neuron analyses. SS performed the immunohistochemistry experiments. DG-R ran the cluster analyses and performed all statistical analyses. All authors read, edited, and approved the final manuscript.

## Funding

This work was supported by NIH NINDS R01 NS104194 and R21 NS118226 (KD), the Edward Jekkal Muscular Dystrophy Association Fellowship (DG-R), and NIH T32 NS121768 (SS).

## Conflict of interest

The authors declare that the research was conducted in the absence of any commercial or financial relationships that could be construed as a potential conflict of interest.

## Publisher's note

All claims expressed in this article are solely those of the authors and do not necessarily represent those of their affiliated organizations, or those of the publisher, the editors and the reviewers. Any product that may be evaluated in this article, or claim that may be made by its manufacturer, is not guaranteed or endorsed by the publisher.

## Supplementary material

Supplementary Figure 1 Correspondence of k and H clusters. Correspondence of *k*-clusters (left squares) and H-clusters (right squares) for each of the 171 Shox2 neurons (cell identification number on the left, Shox2 in black, Chx10 in red). *k*-clusters (left boxes), k1 (purple), *k*2 (blue), *k*3 (yellow), *k*4 (gray), H-clusters (right boxes), H1 (blue), H2 (orange), H3 (yellow), H4 (purple), H5 (green), and H6 (cyan).

Supplementary Table 1 Statistical comparisons of Shox2 and Chx10 neuronal properties presented in Figure 3.

Supplementary Table 2 Statistical comparisons of membrane properties by firing type of Shox2 and Chx10 neurons presented in Figure 4.

Supplementary Table 3 Comparison of cellular properties in neurons separated by k-means clustering presented in Figure 5.

Supplementary Table 4 Comparison of cellular properties in neurons separated by hierarchical clustering presented in Figure 6.
