# Supplementary figures and images for: Identification of adult spinal Shox2 neuronal subpopulations based on unbiased computational clustering of electrophysiological properties
Source: Front Neural Circuits. 2022 Aug 4;16:957084. doi: 10.3389/fncir.2022.957084 (PMC9385948; doi:10.3389/fncir.2022.957084)

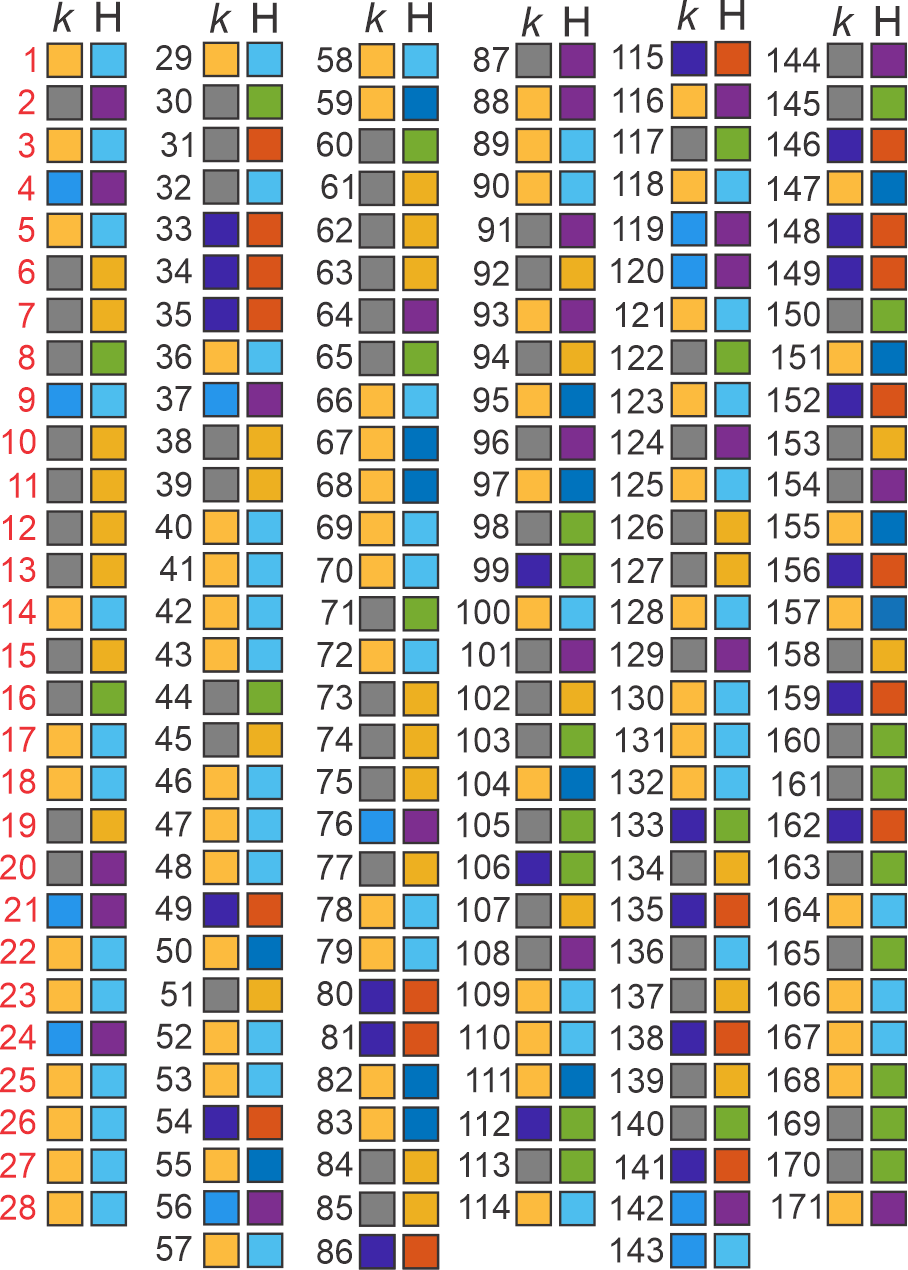

Supplement: Supplementary Figure 1 — Correspondence of k and H clusters. Correspondence of k-clusters (left squares) and H-clusters (right squares) for each of the 171 Shox2 neurons (cell identification number on the left, Shox2 in black, Chx10 in red). k-clusters (left boxes), k1 (purple), k2 (blue), k3 (yellow), k4 (gray), H-clusters (right boxes), H1 (blue), H2 (orange), H3 (yellow), H4 (purple), H5 (green), and H6 (cyan). [file Image_1.TIF]
