# Supplementary material for: Identification of adult spinal Shox2 neuronal subpopulations based on unbiased computational clustering of electrophysiological properties
Source: Front Neural Circuits. 2022 Aug 4;16:957084. doi: 10.3389/fncir.2022.957084 (PMC9385948; doi:10.3389/fncir.2022.957084)
Supplement: Supplementary Table 1 — Statistical comparisons of Shox2 and Chx10 neuronal properties presented in Figure 3. [file Data_Sheet_1.docx]

Supplementary Material

# Supplementary Tables

**Supplementary Table 1.** Statistical comparisons of Shox2 and Chx10 neuronal properties presented in Figure 3.

| Property | Shox2  (n=143) | Chx10  (n=28) | Mann–Whitney (U) or unpaired t-test (t) | p |
| --- | --- | --- | --- | --- |
| Membrane potential [mV] | -48.2 ± 6 | -48.2 ± 5 | U=1972 | 0.9 |
| Input resistance [MΩ] | 711.9 ± 370 | 853.5 ± 337 | U=1417 | *0.01 |
| Time constant [ms] | 41.5 ± 20 | 39.6 ± 14 | U=1982 | 0.9 |
| Capacitance [pF] | 69.2 ± 47 | 50.7 ± 20 | U=1512 | *0.04 |
| Rheobase [pA] | 23.3 ± 13 | 20.8 ± 13 | U=1748 | 0.3 |
| AP Threshold [mV] | -36.3 ± 4 | -35.9 ± 4 | U=1984 | 0.9 |
| AP Half width [ms] | 1.08 ± 0.3 | 1.22 ± 0.3 | U=1339 | *0.005 |
| fAHP duration [ms] | 4.6 ± 1.9 | 5.5 ± 2.5 | U=1434 | *0.01 |
| sAHP duration [ms] | 87.1 ± 65 | 67.1 ± 52 | U=1773 | 0.3 |
| fAHP amplitude | 0.28 ± 0.08 | 0.26 ± 0.06 | U=1855 | 0.5 |
| PIC on voltage [mV] | -47.9 ± 5 | -50.2 ± 5 | t(_169_)=2.3 | *0.02 |
| F/I slope | 0.32 ± 0.2 | 0.24 ± 0.1 | U=1585 | 0.08 |

**Supplementary Table 2.** Statistical comparisons of membrane properties by firing type of Shox2 and Chx10 neurons presented in Figure 4.

| Property/Type of cell | Tonic (n=88) | Initial doublet (n=49) | Initial burst (n=24) | Delay (n=10) | Kruskal-Wallis (H) or one-way ANOVA (F) | p |
| --- | --- | --- | --- | --- | --- | --- |
| Membrane potential [mV] | -47.4 ± 5 | -49.2 ± 7 | -48.0 ± 6 | -49.7 ± 6 | H=3.4 | 0.3 |
| Input resistance [MΩ] | 710 ± 353 | 849 ± 392 | 682 ± 358 | 524 ± 254 | H=9.5 | *0.02 |
| Time constant [ms] | 41.0 ± 20 | 43.3 ± 20 | 37.2 ± 14 | 41.5 ± 25 | H=1.2 | 0.8 |
| Capacitance [pF] | 67.9 ± 48 | 57.8 ± 29 | 63.3 ± 37 | 98.5 ± 75 | H=3.2 | 0.4 |
| Rheobase [pA] | 23.7 ± 14 | 20.3 ± 10 | 23.3 ± 10 | 27.2 ± 14 | H=3.4 | 0.3 |
| AP Threshold [mV] | -36.4 ± 4 | -35.8 ± 4 | -37.3 ± 6 | -33.4 ± 4 | H=5.9 | 0.1 |
| AP Half width [ms] | 1.03 ± 0.3 | 1.13 ± 0.3 | 1.19 ± 0.4 | 1.41 ± 0.3 | H=14.7 | *0.002 |
| fAHP duration [ms] | 4.7 ± 1.8 | 4.5 ± 1.4 | 5.6 ± 3.2 | 5.5 ± 1.3 | H=5.4 | 0.1 |
| sAHP duration [ms] | 52.31 ± 33 | 95.88 ± 52 | 151.9 ± 87 | 80.97 ± 86 | H=51.9 | *0.0001 |
| fAHP amplitude | 0.29 ± 0.08 | 0.26 ± 0.08 | 0.24 ± 0.09 | 0.28 ± 0.06 | H=15.2 | *0.0017 |
| PIC on voltage [mV] | -47.5 ± 5 | -49.2 ± 5 | -51.1 ± 6 | -44.0 ± 4 | F_(3,167)_=7.1 | *0.0002 |
| F/I slope | 0.35 ± 0.20 | 0.28 ± 0.15 | 0.23 ± 0.15 | 0.20 ± 0.17 | H=12.3 | *0.006 |

**Supplementary Table 3.** Comparison of cellular properties in neurons separated by k-means clustering presented in Figure 5.

| Property/Cluster | k1  (n=23) | k2  (n=11) | k3  (n=68) | k4  (n=69) | Kruskal-Wallis (H) or one-way ANOVA (F) | p |
| --- | --- | --- | --- | --- | --- | --- |
| Membrane potential [mV] | -48.8 ± 5 | -46.2 ± 5 | -44.5 ± 3 | -51.8 ± 5 | H=67.3 | *0.0001 |
| Input resistance [MΩ] | 407 ± 160 | 1064 ± 444 | 792 ± 388 | 735 ± 308 | H=33.7 | *0.0001 |
| Time constant [ms] | 56.9 ± 24 | 43.0 ± 14 | 37.9 ± 17 | 38.8 ± 18 | H=14.1 | *0.003 |
| Capacitance [pF] | 148.3 ± 67 | 44.9 ± 18 | 51.8 ± 19 | 56.2 ± 20 | H=59.9 | *0.0001 |
| Rheobase [pA] | 21.9 ± 14 | 16.8 ± 8 | 25.2 ± 15 | 21.9 ± 11 | H=4.5 | 0.2 |
| AP Threshold [mV] | -33.4 ± 4 | -34.7 ± 3 | -36.6 ± 3 | -36.9 ± 5 | F_(3,167)_=5.5 | *0.001 |
| AP Half width [ms] | 1.01 ± 0.3 | 1.58 ± 0.3 | 1.15 ± 0.3 | 1.01 ± 0.3 | H=28.7 | *0.0001 |
| fAHP duration [ms] | 4.1 ± 1.2 | 10.3 ± 2.3 | 4.6 ± 1.6 | 4.2 ± 1.2 | H=24.8 | *0.0001 |
| sAHP duration [ms] | 62.9 ± 46 | 98.3 ± 92 | 90.0 ± 64 | 75.3 ± 61 | H=6.5 | 0.09 |
| fAHP amplitude | 0.32 ± 0.08 | 0.32 ± 0.10 | 0.23 ± 0.06 | 0.30 ± 0.07 | H=41.5 | *0.0001 |
| PIC on [mV] | -45.2 ± 5 | -48.4 ± 6 | -47.8 ± 4 | -49.8 ± 5 | F_(3,167)_=5.8 | *0.0009 |
| F/I slope | 0.36 ± 0.19 | 0.29 ± 0.15 | 0.29 ± 0.21 | 0.29 ± 0.10 | H=3.0 | 0.1 |

**Supplementary Table 4.** Comparison of cellular properties in neurons separated by hierarchical clustering presented in Figure 6.

| Property/  Cluster | H1 (n=15) | H2 (n=20) | H3 (n=32) | H4 (n=25) | H5 (n=27) | H6 (n=52) | Kruskal-Wallis (H) or one-way ANOVA (F) | p |
| --- | --- | --- | --- | --- | --- | --- | --- | --- |
| Membrane potential [mV] | -46.5 ± 3 | -49.1 ± 6 | -55.7 ± 5 | -46.7 ± 4 | -48.2 ± 3 | -44.3 ± 3 | H=82.6 | *0.0001 |
| Input resistance [MΩ] | 812 ± 222 | 414 ± 174 | 675 ± 257 | 1092 ± 371 | 736 ± 464 | 788 ± 296 | H=42.4 | *0.0001 |
| Time constant [ms] | 46.8 ± 17 | 59.0 ± 25 | 39.3 ± 15 | 37.9 ± 12 | 42.4 ± 23 | 34.8 ± 16 | H=20.1 | *0.001 |
| Capacitance [pF] | 60.3 ± 23 | 153.5 ± 71 | 61.3 ± 21 | 37.9 ± 15 | 63.7 ± 23 | 52.0 ± 17 | H=70.7 | *0.0001 |
| Rheobase [pA] | 15.6 ± 7 | 19.3 ± 12 | 19.5 ± 9 | 17.7 ± 7 | 26.3 ± 12 | 25.5 ± 15 | H=14.2 | *0.02 |
| AP Threshold [mV] | -36.9 ± 3 | -33.5 ± 4 | -38.0 ± 6 | -36.6 ± 5 | -34.7 ± 3 | -36.6 ± 3 | H=23.6 | *0.0003 |
| AP Half width [ms] | 1.04 ± 0.2 | 1.07 ± 0.3 | 1.05 ± 0.3 | 1.29 ± 0.3 | 0.90 ± 0.2 | 1.18 ± 0.3 | H=24.1 | *0.0002 |
| fAHP duration [ms] | 3.3 ± 1.7 | 4.2 ± 1.2 | 4.1 ± 1.0 | 7.3 ± 2.9 | 3.9 ± 1.3 | 5.1 ± 1.5 | H=42.6 | *0.0001 |
| sAHP duration [ms] | 72.2 ± 35 | 82.3 ± 70 | 92.2 ± 64 | 77.3 ± 70 | 53.6 ± 41 | 67.8 ± 61 | H=9.2 | 0.1 |
| fAHP amplitude | 0.18 ± 0.07 | 0.30 ± 0.07 | 0.26 ± 0.06 | 0.31 ± 0.08 | 0.36 ± 0.06 | 0.25 ± 0.05 | H=59.8 | *0.0001 |
| PIC on [mV] | -46.8 ± 3 | -45.8 ± 5 | -52.0 ± 4 | -50.9 ± 5 | -44.9 ± 3 | -47.9 ± 4 | F_(5,165)_=11.8 | *0.0001 |
| F/I slope | 0.54 ± 0.17 | 0.33 ± 0.19 | 0.28 ± 0.11 | 0.41 ± 0.21 | 0.32 ± 0.18 | 0.18 ± 0.12 | H=48.7 | *0.0001 |
